# Accounting for Persistence in Tests with Linear Ballistic Accumulator Models

**DOI:** 10.1017/psy.2025.10026

**Published:** 2025-06-16

**Authors:** Jochen Ranger, Sören Much, Niklas Neek, Augustin Mutak, Steffi Pohl

**Affiliations:** 1 Department of Psychology, Martin-Luther-Universität Halle-Wittenberg, Halle, Germany; 2 Wilhelm Wundt Institute for Psychology, Leipzig University, Leipzig, Germany; 3 Faculty of Education and Psychology, Freie Universität Berlin, Berlin, Germany; 4 Faculty of Education and Psychology, University of Zagreb, Zagreb, Croatia

**Keywords:** item response theory, linear ballistic accumulator, rapid guessing, response time

## Abstract

In this article, we propose a series of latent trait models for the responses and the response times on low stakes tests where some test takers respond preliminary without making full effort to solve the items. The models consider individual differences in capability and persistence. Core of the models is a race between the solution process and a process of disengagement that interrupts the solution process. The different processes are modeled with the linear ballistic accumulator model. Within this general framework, we develop different model variants that differ in the number of accumulators and the way the response is generated when the solution process is interrupted. We distinguish no guessing, random guessing and informed guessing where the guessing probability depends on the status of the solution process. We conduct simulation studies on parameter recovery and on trait estimation. The simulation study suggests that parameter values and traits can be recovered well under certain conditions. Finally, we apply the model variants to empirical data.

The motivation to take a test is an important determinant of the test result (Silm et al., 2020). Test-taking motivation is an internal state that drives test takers to engage in the test and pursue good test results (Bandhu et al., 2024). It determines the intensity by which test takers work and the time they are willing to spend on the test (Baumert & Demmerich, 2001; Eklöf, 2010; Knetka, 2017). The intensity depends on the extent to which mental resources are mobilized and assigned to a task (Ranger & Kuhn, 2014; Wenger & Gibson, 2004). The willingness to spend time on the test determines the maximal time, a test taker is disposed to invest into the test and the single items. In this article, we focus on the willingness to spend time on the test and ignore the second facet of test taking motivation.

It is well known that in achievement tests, the test results do not only depend on the capability of the test takers, but also on their motivation to take the test. Recent findings suggest that the time dedicated to the items has a strong relation to the test score (Cheyette & Piantadosi, 2024). In fact, the importance of the time spent on the solution process has been acknowledged at the very beginning of psychometrics (Thurstone, 1937). Findings also suggest that test takers differ systematically in the amount of time they invest into a test. This characteristic of a test taker may be represented by a latent trait that we denote as persistence. Test takers with a low value of persistence are not willing to spend much time on the items and potentially quit working prematurely. The persistence of test takers has been conceptualized differently in the literature. On the one hand, it has been conceptualized as a stable trait that determines the time investment in the single items throughout the test (Goldhammer et al., 2017). In this conceptualization, the perspective is on the general level of the time investment. On the other hand, it has been conceptualized as the maintenance of engagement throughout the test (Nagy & Robitzsch, 2021). In this conceptualization, the perspective is on the change or rather the absence of it over the test. In the article, we understand persistence in the first sense, as the general tendency of a test taker to invest time into the single items. A formal definition of persistence in mathematical terms is given in the next section.

The influence of test taking motivation on the test results implies that the test scores of the test takers do not only reflect their level of ability, but also their level of persistence. This is problematic in case ability is the target trait of the assessment. The joint influence of ability and persistence on the test results has to be disentangled by a measurement model that considers both quantities.

## 1 Latent trait models with persistence components

The responses of test takers in tests are often modeled with item response theory models (Embretson & Reise, 2000). Standard item response theory models assume that the probability to solve an item depends on the difficulty of the item and the effective ability of a test taker. The effective ability, however, confounds capability and persistence as highlighted in the previous section. While extensions like bifactor models (Gibbons & Hedeker, 1992) or non-compensatory item response models (DeMars, 2016; Suh & Bolt, 2010) might to some extend capture the effects of persistence on test taking in further latent traits, they cannot represent persistence as a variable related to the invested time directly. This requires models for responses and the response times. Models that account for individual differences in persistence can be classified into two classes, mixture models and race models.

Mixture models for responses and response times were proposed for low stakes tests or tests with a strict time limit in order to model rapid guessing (Alós-Ferrer, 2018; Lu et al., 2020; Meyer, 2010; Molenaar et al., 2018; Nagy & Ulitzsch, 2022; Ulitzsch et al., 2022; Wang & Xu, 2015). Here, we focus on their application to low stakes tests. In their simplest form, the mixture models assume two different modes of responding, a fast one where test takers guess rapidly and a slow one where test takers respond regularly. Fast guesses are responses with a very low investment of time. They are construed as the result of random responding and do not reflect a test taker’s capability. Regular responses, on the other hand, are responses with a high investment of time. They are supposed to reflect the capability of the test taker. In mixture models, individual differences in persistence are captured in the propensity to respond with one of the two response modes. Test takers with low persistence have a high frequency of rapid guessing.

A different conceptualization of persistence is made by race models. Race models assume a race between the response process and a timer that interrupts the response process. When the response process is interrupted prematurely, the test taker responds either incorrectly, omits the item or selects a response by guessing. First versions of race models were proposed by Roskam (1987) and Glickman et al. (2005), although these authors did not refer to persistence. Lee and Ying (2015) and Lu and Wang (2020) proposed mixture cure-rate models for responses and response times where the response process is censored by a censoring time. In their models, the censoring time is not related to characteristics of the test takers. Ranger and Kuhn (2014), on the other hand, assumed a race between information processing and disengagement and related both processes to latent traits. Hawkins and Heathcote (2021) proposed a model where different response options race against a censoring time. In the race models, the persistence of a test taker is represented by a latent trait that is related to the censoring time. This allows for individual differences in the time spent on a task. Test takers with a very low level of persistence tend to give responses fast and prematurely. But note that also test takes with a high level of persistence may interrupt processing in case an item is very time demanding.^1^

The overview illustrates that the mixture models and the race models make different assumptions about the response process. In specific, they differ in their assumptions about persistence, guessing and the generation of incorrect responses. We discuss these aspects in the following.

Mixture models distinguish two modes of responding, a fast one that leads to a rapid guess and a slow one that leads to a regular response. Individual differences in persistence manifest in the frequency by which a test taker is in a particular response mode. In the fast response mode, however, there are no individual differences with respect to the solution probability and the response time distribution. This assumption contradicts the observation that even test takers with reduced engagement differ systematically in the time they invest into the items (Cheyette & Piantadosi, 2024). Persistence is thus more than just the propensity to respond by rapid guessing. The race models, on the other hand, relate the maximal time, a test taker is willing to spend on an item, to a latent trait of a test taker. This makes them more adequate for low-stakes tests where test takers differ gradually in their test taking motivation. Race models, however, are less adequate for speeded tests where the time spent on an item changes rapidly when test takers run out of time.

In the mixture models, the responses are either regular responses or random guesses. A random guess is a guess with a fixed guessing probability that may differ over items, but not over test takers. This implies that guessing is not related to the capability of a test taker or the actual progress a test taker has made when working on an item. Guesses, however, are rarely entirely random (Noventa et al., 2024). Evidence suggests that test takers use partial knowledge when guessing. Partial knowledge is incomplete knowledge that improves the probability of a successful guess (Burton, 2002). Partial knowledge may be used for a comparative evaluation of all response options (Suh & Bolt, 2010) or for the elimination of distractors in single-choice tests (Lau et al., 2011). This suggests the existence of two forms of guessing behavior, rapid guessing and informed guessing (Guthrie et al., 2020). In item response theory, informed guessing is modeled by relating the guessing probability to the capability of a test taker (San Martin et al., 2006) or by a sequential process where distractors are eliminated (Bechger et al., 2005). Informed guessing cannot be accounted for by the mixture models reviewed above. The race models are deficient in this aspect also. They either assume no guessing or random guessing.

In the race models, the incorrect responses are generated by an interruption of the response process. This implies that the solution probability increases with the time spent on an item. This assumption may not hold in practice as findings suggest that incorrect responses can be generated actively (Duncan, 1974; Stupple et al., 2017). This happens in case a test taker has a misconception of the problem and believes that an incorrect solution is correct (Frary, 1980). A misconception is different from partial knowledge or lack of understanding (Lau et al., 2011) as it generates incorrect responses irrespective of the given response options in a single-choice test and lowers the solution probability beyond what is expected by chance (Sadler, 1998; Suh & Bolt, 2010). A detailed analysis of the misconceptions held by the test takers requires single-choice tests with tailored distractors and an analysis with nominal response models (Sadler, 1998), Bayesian networks (Lee & Corter, 2011) or cognitive diagnosis models (de la Torre, 2009). Race models with two accumulators representing information processing and disengagement cannot account for the effect of misconceptions. The mixture models reviewed above are better suited for misconceptions. Although they are not capable to diagnose the specific kind of misconception, they allow for incorrect responses that are not caused by guessing.

In this article, we address some of the issues raised above. We propose six models for the responses and response times on tests that account for individual differences in the time the test takers are willing to spend on the items. The models are based on the linear ballistic accumulator model (Brown & Heathcote, 2008; Rouder et al., 2015). The proposed models differ in the number of the accumulators (two/three) and their assumption about guessing (none/random/informed). By considering informed guessing, we cover the twilight zone between rapid guessing on a total random basis and regular responding. The article is structured as follows. First, we present the different models. Then, we report the results of a simulation study on the recovery of the model parameters and the latent traits. Thereafter, we apply the proposed model to empirical data. The article ends with a discussion.

## 2 Log-normal race models for persistence in tests

In psychometrics, there has been increased interest in psychometric process models (Batchelder, 2007). Psychometric process models are measurement models that are derived from a mathematical description of the response process and promise a more profound characterization of the test takers than item response models that simply describe test takers in terms of their effective ability to solve items (van der Maas et al., 2011). In the following, we propose a process model on basis of the linear ballistic accumulator model (Brown & Heathcote, 2008; Rouder et al., 2015). We chose the linear ballistic accumulator model for its very good trade-off between simplicity and flexibility. Besides, it has an extendible modular structure and is interpretable in terms of a log-normal race model. Throughout the article, we assume that a test consists of a series of items and that the responses and the response times have been recorded. The response format of the items may be free or single-choice. Responses are scored as either correct or incorrect.

In the linear ballistic model, the responses are generated by a race of different accumulators. In a first series of models (Model A1–A3), we assume two accumulators. The first accumulator represents the progress of a test taker toward solving an item and the second accumulator his tendency to disengage. The first accumulator triggers the correct response. The second accumulator triggers either an incorrect response (Model A1), a random guess (Model A2) or an informed guess (Model A3). In a second series of models (Model B1–B3), we assume three accumulators. As in Model A1–A3, the first and second accumulator in Models B1–B3 represent the progress and the disengagement, respectively. We additionally assume a third accumulator that represents the tendency to respond incorrectly. The first accumulator triggers a correct response, the third accumulator an incorrect response and the second accumulator an incorrect response (Model B1), a random guess (Model B2) or an informed guess (Model B3). An overview of the different models considered in the article is given in Table 1. A more thorough description follows in the next two sections.

**Table 1:** Overview of the different models for persistence

Class	Variant	Accumulators	Guessing
Model A	A1	Progress / Disengagement	None
	A2	Progress / Disengagement	Random
	A3	Progress / Disengagement	Informed
Model B	B1	Progress / Disengagement / Misinformation	None
	B2	Progress / Disengagement / Misinformation	Random
	B3	Progress / Disengagement / Misinformation	Informed

### 2.1 Models of Class A

In Models A1–A3, we assume that the response process can be described by two accumulation processes. The first accumulator represents the progress toward the solution that was made when working on an item. The progress toward the solution increases linearly over time. The drift rate 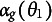 of the progress of a test taker in item *g* is a log-linear function of the test taker’s capability 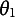 and two item parameters 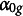 and 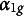 : (1)

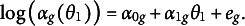



The value of 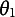 ( 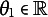 ) quantifies the level of capability a test taker has. Item parameter 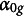 ( 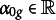 ) is an intercept parameter that determines the drift rate of a reference test taker with trait level 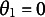 . Item parameter 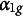 ( 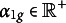 ) is a discrimination coefficient that determines the influence the capability has on the drift rate. The residual 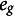 ( 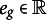 ) is a random variable that represents all additional influences on the accumulation process that are unrelated to the capability of the test taker. The role of 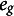 is similar to the role of the specific influences in factor analysis.

As a consequence of the linear ballistic accumulation, the progress 
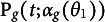
 of a test taker with capability 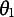 and drift rate 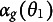 in item *g* at point of time *t* is: (2)

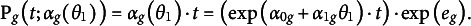



Equation (2) implies that not all test takers with the same level of capability 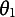 make the same progress in item *g*. The individual progress of the test takers fluctuates around the typical progress 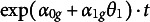 due to the random influence 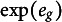 . This random influence accounts for the noise of the information accumulation. In the following, we assume that 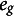 is a normally distributed random variable with expectation of zero and standard deviation of 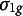 . This implies that the progress at each point of time has a log-normal distribution with scale parameter 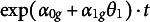 and shape parameter 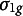 in case the latent trait is considered as fixed.

When processing an item, test takers make progress toward the solution, but also lose their engagement to work on the task. We assume a second linear ballistic accumulator that represents the tendency to disengage from the task. The disengagement tendency increases linearly over time with a second drift rate. We assume that the second drift rate is related to the persistence 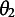 of a test taker and two item parameters 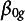 and 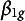 via the log-linear model: (3)

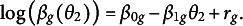



The value of 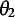 ( 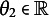 ) quantifies the persistence a test taker has. The item parameters 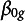 ( 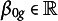 ) and 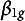 ( 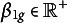 ) can be interpreted in parallel to 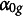 and 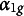 . In contrast to Equation (1), the contribution of 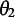 to 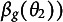 is negative. This justifies the interpretation of 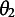 in terms of persistence as high values of 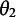 correspond to low levels of disengagement. The random variable 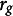 is a residual that represents all additional influences on the disengagement process.

As a consequence of the linear ballistic accumulation, the disengagement tendency 
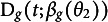
 of a test taker with persistence 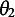 and drift rate 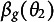 in item *g* at point of time *t* is: (4)

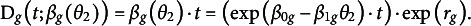



The residual 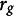 represents the noise of the accumulation process. It is assumed to be distributed according to a normal distribution with expectation of zero and standard deviation of 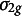 . This implies that the disengagement tendency at each point of time has a log-normal distribution with scale parameter 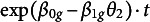 and shape parameter 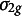 in case the latent trait is considered as fixed.

For both accumulators, we assume two critical thresholds. The first threshold 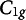 represents the progress that has to be made in order to solve an item. As soon as the momentary progress of a test taker 
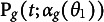
 exceeds 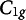 , a correct response can be given. This happens at the solution time 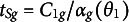 . As the log-transformed solution time 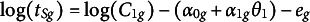 is a linear function of capability and the residual 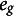 , the solution time has a log-normal distribution. The second threshold 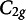 represents the level of disengagement at which test takers stop working. This happens at the disengagement time 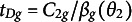 . As the log-transformed disengagement time 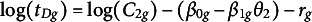 is a linear function of persistence and the residual 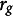 , the disengagement time is likewise log-normally distributed.

The response and the response time in an item are the result of a race between the two accumulators (Rouder et al., 2015). The observed response time 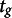 is the hitting time of the faster accumulator. It is the minimum of the solution time and the disengagement time 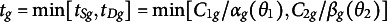 . The response 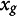 depends on which accumulator reaches its threshold first. In case the accumulator representing progress wins, the response is always the correct solution. In case the accumulator representing disengagement wins, the response depends on the version of the model. In a first version of the model (Model A1), the test takers always respond incorrectly. In a second version of the model (Model A2), the test takers guess randomly. In this case, the guessing probability is an item dependent quantity 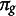 that does not depend on the latent trait of a test taker. In a third version of the model (Model A3), the guessing probability depends on the progress that was made up to 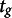 . We assume that the interval 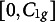 represents the continuum from random guessing to a correct solution. When 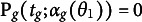 , there was no progress and the solution probability is on the level 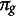 of a random guess. When 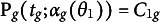 , the test taker has made enough progress in order to solve the item. In this case, the solution probability is 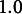 . For all other levels of progress, the guessing probability is determined by the linear interpolation: (5)

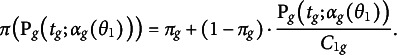



**Figure 1:**
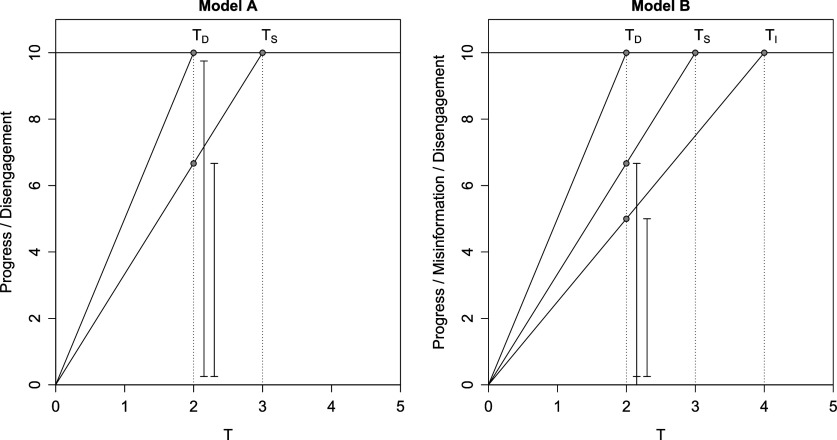
Illustration of the assumed response process for variant A and variant B of the linear ballistic accumulator model.

An example of the assumed response process is given in Figure 1, left plot. The line from 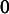 to 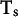 visualizes the progress of a test taker with drift rate of 
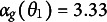
 . This drift rate implies that the test taker requires a solution time of 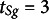 in order to make enough progress ( 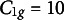 ) to solve the task. The line from 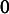 to 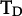 visualizes the disengagement tendency of a test taker with drift rate 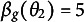 . The disengagement accumulator hits the threshold 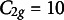 at the disengagement time 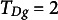 and interrupts the solution process before the solution has been found. In Model A1, the test taker would respond incorrectly. In Model A2, the test taker would guess randomly with a fixed guessing probability of, for example, 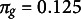 . In Model A3, the test taker would guess on basis of the progress that was made up to the time 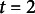 . As 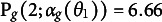 , the guessing probability is 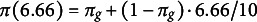 , which would be 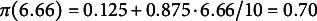 when the probability of a random guess is 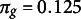 .

From the distributional assumptions about the residuals, the joint distribution of the response and the response time in an item can be derived. In order to save space, we just give a short sketch of the derivation. The solution time and the disengagement time are determined by the thresholds and the drift rates. The solution time, for example, is 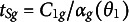 . For fixed latent traits, the drift rates are log-normally distributed. The inverse value of a log-normal random variable is likewise log-normally distributed. The solution and the disengagement time are thus log-normally distributed. The response time in Model A is the minimum of the solution and the disengagement time. Both times are log-normally distributed and independent as the residual terms 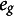 and 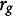 are independent. The density function of the first hitting time of the winning accumulator is thus the product of a log-normal density function with the distribution function of a log-normal distribution. The observed responses are determined by the winning accumulator. In Model A1, the response is always incorrect when the disengagement accumulator wins. In Model A2, the response is set by a guessing process that acts independently from the response time with a fixed guessing probability. In Model A3, the guessing probability depends on the relation of the progress to the threshold 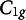 at the disengagement time. The progress at the disengagement time, given that the disengagement accumulator wins, is distributed according to a truncated log-normal distribution. The guessing probability is the ratio of the accumulated progress and the threshold 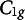 . It likewise has a truncated log-normal distribution. The subdensity function of the disengagement time is divided between the correct and incorrect response according to the potential guessing probability. This requires integration over the truncated log-normal distribution. The implied subdensities of Model A1–A3 can be found in Section S1 of the Supplementary Material.

### 2.2 Models of Class B

In Models A1–A3, an item can always be solved when the persistence is sufficiently high. This assumption is plausible for simple tasks (e.g., simple calculations), but implausible for more complex tasks (e.g., items on crystallized intelligence). To overcome this limitation, we extend the models by a third accumulator that represents the progress toward an incorrect solution, a quantity we denominate as misinformation in the following. The incorrect solution subsumes all systematically wrong responses that are due to a misconception of the problem. The misinformation in item *g* increases linearly over time with a drift rate that depends on a third latent trait 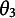 , namely the error-proneness of a test taker, and two item parameters 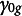 and 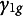 via the log-linear model: (6)

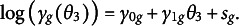



The value of 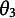 ( 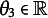 ) quantifies the proneness of a test taker toward an incorrect solution. The item parameters 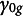 ( 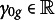 ) and 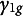 ( 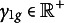 ) can be interpreted in parallel to the corresponding item parameters in Models A1–A3. The quantity 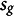 is a random variable that generates noise in the accumulation process. It represents all further influences on the accumulation process. It is assumed to have a normal distribution with expectation of zero and standard deviation 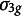 . As a consequence, the misinformation 
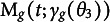
 of a test taker with drift rate 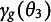 in item *g* is a linear function of the time spent on the item: (7)

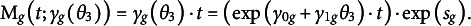



The observed response time is the result of a race of the three accumulators 
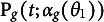
 , 
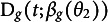
 and 
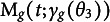
 toward accumulator specific thresholds 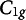 , 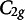 , and 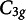 . The accumulator that first hits its threshold determines the observed response and response time. If the accumulator 
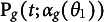
 representing progress wins, the correct response is given. If the accumulator 
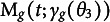
 representing misinformation wins, an incorrect response is given. If the accumulator 
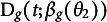
 representing disengagement wins, the response depends on the version of the model. In Model B1, the response is always incorrect. In Model B2, the test takers guess randomly with a fixed guessing probability of 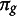 that does not depend on the test takers’ capabilities or any accumulator. In Model B3, the test takers make an informed guess. The response is correct when 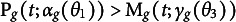 and incorrect when 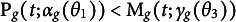 . Note that the guessing process is determined by the relation of the momentary progress to the momentary misinformation. A test taker selects the response that has the strongest support or that is hold most actively in mind. This guessing process is different to the informed guessing process in Model A3, which is independent of alternative response options. The observed response time is always the minimum of the three hitting times.

An example of the assumed response process is given in Figure 1, right plot. In the example, the test taker has the drift rates 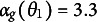 , 
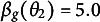
 , and 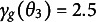 . The line from 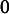 to 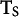 depicts the progress, the line from 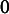 to 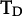 the disengagement tendency and the line from 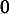 to 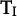 the misinformation. The disengagement accumulator hits its threshold 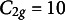 at the disengagement time 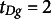 and interrupts the response process prematurely. The progress and misinformation of the test taker at this point of time are 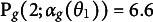 and 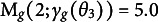 , respectively. In the example, the response time is 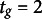 . For Model B1, the observed response would be incorrect. For Model B2, the observed response would be the result of a random guess with a fixed guessing probability of, for example, 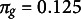 . In Model B3, the response would be correct as the progress is larger than the misinformation when responding.

The distributional assumptions about the residuals determine the joint distribution of the response and response time in an item. In order to save space, we just sketch how the density functions can be derived. The response time is determined by the hitting times of each accumulator as the minimum of three independent log-normally distributed random variables. Its distribution follows from standard results on order statistics. The probability of a response is determined as in Models A1–A3 in case the accumulator representing progress or misinformation wins. The case that the disengagement accumulator wins, has to be treated differently. In Model B1, the response is directly determined by the winning accumulator. In Model B2, the response is determined by a guessing process with fixed guessing probability that does not dependent of the response time or the remaining accumulators. In Model B3, the response is determined by the accumulator with the higher level. The progress and misinformation at time *t*, given that both quantities are below their threshold, are distributed according to two independent truncated log-normal distributions. The guessing probability is thus the probability that a truncated log-normally distributed random variable exceeds another one. The density function of the response time 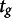 is then divided between the correct and incorrect response according to the potential values of the guessing probability. The implied subdensities of Model B1–B3 can be found in Section S1 of the Supplementary Material.

### 2.3 The joint distribution of the responses and response times in a test

Model A1–A3 and Model B1–B3 specify the distribution of the response and response time in a single item conditional on the trait levels of a test taker. We denote the subdensity function of this distribution as 
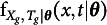
 ; see Section S1 of the Supplementary Material for more details. The vector 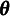 represents the latent traits of a test taker, which are 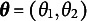 in Model A and 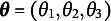 in Model B. Item parameters are not mentioned explicitly. In order to derive the joint distribution of the responses 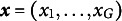 and response times 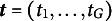 in a test of *G* items, we assume conditional independence. This is the standard assumption in item response theory (Embretson & Reise, 2000). Conditional independence means that responses and response times in different items are independent when conditioning on the latent trait. The joint distribution of the responses and response times 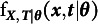 then factors into the product of the item specific subdensities: (8)

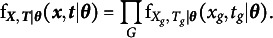



Equation (8) is the distribution of the responses and response times conditional on specific values of the latent trait. The marginal distribution is the distribution of the responses and response times when test takers are sampled randomly. The marginal distribution 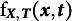 follows from the conditional distribution 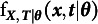 when integrating over the distribution of the latent traits 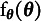 in the population of the potential test takers the actual test takers were sampled from. In item response theory, it is standard practice to assume that the latent traits are standardized multivariate normally distributed random variables with unrestricted correlation matrix 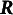 . The normal distribution is unimodal and symmetric and for this reason considered as an adequate representation of the distribution of human characteristics (Sartori, 2006). In low stakes tests, however, there is sometimes an imbalance between test takers with low and high persistence. This requires skewed distributions. One option in this case is to use a mixture of two multivariate normal distributions (McLachlan & Peel, 2000). Irrespective of the distribution one deems most adequate, the scale of the latent traits has to be fixed. We here consider standardization of the latent variables, but other identification restrictions are possible as well.

## 3 Strengths, limitations, and relations to alternative models

The proposed models make precise assumptions about the data generating process. In the following, we discuss these assumptions. We also compare the model to models that have been proposed before. Specifically, we address the following aspects: Interpretation of accumulators, ballistic accumulation, linear accumulation, conditional independence of accumulators, response specific accumulators, nature of latent traits, and informed guessing.

### 3.1 Interpretation of accumulators

Process models in general and the proposed variants of the LBA model in specific are models of the response process on a high abstract level (Wagenmakers, 2009). They are general models which represent the progress or accumulation of misinformation on a continuum. The accumulators are not directly related to possible substages of the response process, knowledge states or the neural substrate underlying problem solving. This is different to cognitive diagnostic modeling or knowledge structure analysis where detailed assumptions about the tasks and their demands are made (Heller, 2023; Noventa et al., 2024). Abstract models like the ones proposed in this article have the advantage of being generally applicable as no theory about the tasks is necessary.

### 3.2 Ballistic accumulation

In the proposed models, accumulation is ballistic as random variation impacts the whole path of the accumulator in the same way. The progress at a specific point of time, for example, is modeled as 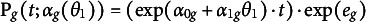 . The overall effect of all random noise in information accumulation is summarized in the residual 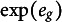 . This is different to the diffusion model (Ratcliff & McKoon, 2008) where the product of drift rate and time is perturbed at any point in time by momentary fluctuations. The diffusion model, however, was originally proposed for simple perceptual decisions where the random fluctuations were supposed to account for spontaneous neural activity. Spontaneous fluctuations are less plausible in more complex tasks. We consider ballistic accumulation as a reasonable simplification of the real progression of information accumulation or disengagement. Modeling momentary fluctuations would increase the model’s complexity disproportionately.

### 3.3 Linear accumulation

In the proposed model, we assume a linear increase of progress, disengagement, and misinformation over time. Accumulators are always increasing as the drift rates are restricted to be positive. This implies that each accumulator will eventually hit its threshold. While this is realistic for disengagement, it might be problematic for the accumulator representing progress as some test takers might be incapable to solve an item even in infinite time. Replacing the log-link in Equation (1) with an alternative link (e.g., an identity link) would resolve this issue. The increase of the accumulators over time is modeled by a linear function. This is similar to the diffusion model (Ratcliff & McKoon, 2008; van der Maas et al., 2011) where the expected preference formation is also a linear function of time. Linear accumulation is a strong assumption that excludes acceleration or deceleration. The assumption of linearity is avoided in the race model of Ranger & Kuhn (2014), where progress and disengagement accumulation are modeled by splines. While this might be more realistic, it complicates the extension to partial guessing. Recently, Much et al. (2022) proposed an accumulation model where progress increases monotonically to an upper asymptote. Test takers stop accumulating in case the growth rate drops below a critical threshold. This model avoids the linearity assumption, but cannot be interpreted in terms of persistence directly.

### 3.4 Conditional independence of accumulators

In the present model, the process of information, misinformation, and disengagement accumulation are unconditionally related by a possible interdependence of the latent traits underlying the accumulators. The residual terms 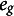 , 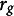 and 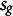 are assumed to be independent. This implies that the typical paths 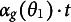 , 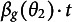 and 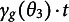 might be related via the correlations of the latent traits, but not the specific deviations. Untypically slow progress in an item that is not explained by a person’s capability has therefore no impact on the development of disengagement. The models thus separate the solution process from the process of motivation, unlike models where both processes are interrelated (e.g., Cisek et al., 2009; Much et al., 2022). Although the assumption made in our model is strong, it is weaker than the assumptions that were made in the cure mixture model of Lee and Ying (2015). In their model, the solution process is interrupted by a completely independent censoring time. The assumption of conditional independence in the proposed model could be relaxed like in the model of Ranger et al. (2015) where two competing accumulators are related by a bivariate normal copula. This model, however, was not intended to model persistence. Our model is in-line with the motivation of the hierarchical modeling framework for responses and response times (van der Linden, 2007). In the hierarchical modeling framework, single components of the response process are only related on a higher level through the latent traits. This is justified by the assumptions that test taker form their test taking motivation before taking a test and do not change it anymore when working.

### 3.5 Response specific accumulators

In the proposed model, we score responses as either correct or incorrect. Different incorrect responses, e.g., by different distractor choices in a single-choice test, are not distinguished. The fusion of different types of wrong responses into an overall category is not unusual in psychometric process models. It is, for example, also made in the psychometric extensions of the diffusion model by van der Maas et al. (2011). The assumption can be justified by further assumptions. Given that there are several accumulators for incorrect responses, only the incorrect accumulator with the minimal hitting time is relevant for the response and response time. Representing several accumulators by one requires that the minimum of the hitting times of all incorrect accumulators is log-normally distributed and depends on just one latent trait. This implies that the original distributions of all incorrect accumulators were such that the minimum of possibly correlated random draws is log-normally distributed. If this assumption appears too strong, it is possible to introduce accumulators for each specific wrong response as it was done by Bunji and Okada (2022) in their model for personality tests; see also Hawkins and Heathcote (2021). Assuming response-specific accumulators, however, is problematic in achievement tests as even in single-choice items, it is not known which response options are actually considered by a test taker (Vigneau et al., 2006).

### 3.6 Nature of latent traits

In Models B1–B3, the performance in a test is related to three latent traits, one representing the persistence of a test taker and the other two his/her capability and error-proneness. Capability and error-proneness are conceived as two distinct, but possibly related quantities. This is similar to the race model of Ranger et al. (2015), but differs, for example, from the diffusion model where the two traits are interwoven. Large correlations between the traits could be interpreted in terms of a higher order factor model (parallel measures assumed). Alternatively, as in Ranger et al. (2015), one could reparametrize capability and error-proneness as 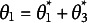 and 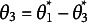 . Here, the first trait 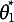 denotes the overall speed of a test taker and 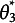 the preference of one response option over the other. The interpretation of 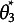 would then be similar to the interpretation of the drift rate in the diffusion model.

The trait that represents the persistence of a test taker is assumed to be constant over the test. We assume that the effort of a test taker is determined by the value of the task which does not change during test taking. This is similar to the mixture model of Meyer (2010) where test takers are characterized by a fixed engagement level. In this aspect, our model differs from models that assume systematic changes in performance during test taking (e.g., Fox & Marianti, 2016; Mutak et al., 2024; Ranger et al., 2023), switches between engagement levels during the test (e.g., Molenaar et al., 2018; Ulitzsch et al., 2022) or changes in persistence when test takers run out of time (e.g., Wang & Xu, 2015); note, however, that some change is possible in our model. General declines in persistence or item position effects can be absorbed in the intercept parameter 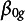 , which is item dependent. Changes in the time investment from item to item are generated by the random variation of the disengagement accumulator. The relation of the discrimination parameter 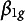 to the residual variance 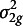 determines the variation of the invested time within the test and its correlation between items. This allows for a purely random process in each item when the discrimination coefficient is zero and an almost determined process then the discrimination coefficient is very high. In this aspect, the proposed models are more flexible than the mixture models for test engagement.

### 3.7 Informed guessing

In Models A3 and B3, the probability to solve an item by guessing depends on the progress that was made by a test taker till disengagement. This implies that test takers with higher levels of capability or longer processing times have a higher probability to guess correctly. This is in contrast to guessing in the mixture models for rapid guessing or the conception of guessing in the three-parameter logit model (von Davier, 2009). In these models, test takers guess on a random basis with the same guessing probability, irrespective of their ability level or the time they spent on an item. Random guesses are considered in our models as informed guesses on a very low information level, which is a result of a very short processing time or a low level of capability. Model A3 and Model B3 also differ from item response models with ability-based guessing. In models for ability-based guessing, the guessing probability either directly depends on a test takers ability (San Martin et al., 2006) or on a second latent trait that could, for example, reflect test-wiseness (DeMars, 2016). In the proposed models, the dependency of the guessing probability on the capability of a test taker is mediated through the actual progress that was made. This implies that the solution probability is also not a direct function of time as, for example, in Bolsinova and Molenaar (2018) or Wang and Hanson (2005). In item response theory, partial knowledge has also been modeled with the Nedelsky model (Bechger et al., 2005) where partial knowledge is used for excluding distractors in single-choice response sets. The response is then selected among the remaining distractors randomly. Such a form of guessing could be integrated in the proposed model when specific levels of progress would be associated with specific subsets of distractors. The linear dependency of the guessing probability (or its logit) on the actual progress in Model A3 is a strong assumption. While it is plausible that the attraction of the correct response should increase with the knowledge level, the linearity of the relation cannot be justified from the model alone. A justification would require a precise model for the knowledge structure assessed by the items. This is typically not available. In Model B3, the guessing probability depends on a comparison of the evidence accumulated for the correct and the incorrect response. This guessing process has some resemblance to guessing in the diffusion model (Ratcliff, 1988).

## 4 Parameter estimation

The item parameters of the model are not uniquely identified as in a latent accumulation process, the scale of the accumulators is arbitrary; note that the first hitting times are not affected when the drift rate and the threshold of an accumulator are shifted or multiplied by the same amount. This is not specific for the proposed variants of the LBA model, but occurs in the graded response model or the diffusion model also. In order to identify the model, one parameter must be fixed (Brown & Heathcote, 2008). Here, we set all thresholds to an arbitrary constant. As this is an identification restriction, it sets the scale for the accumulators and does not have any consequences on model fit.^2^

Item parameters can be estimated by marginal maximum likelihood estimation. This requires assumptions about the distribution of the latent traits. In the following, we assume that the latent traits are multivariate normally distributed with unrestricted correlation matrix 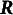 , although other distributions would be possible. The item parameters of the model are referred to as 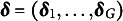 . Here, 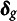 represents the parameters in the *g*-th item, that is 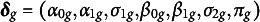 in Model A and 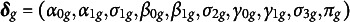 in Model B. The marginal log-likelihood function is a function of the model parameters. It is defined as: (9)

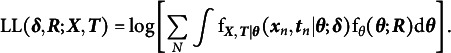



In Equation (9), the matrices 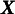 and 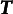 contain the responses and response time pattern 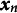 and 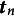 of 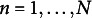 test takers. Function 
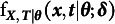
 is the subdensity of the conditional joint distribution of the responses and response times (Equation (8)) with item parameters mentioned explicitly. Function 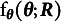 is the density function of the standard multivariate normal distribution with covariance matrix 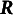 . In marginal maximum likelihood estimation, one determines those parameter values that maximize the marginal likelihood function. This includes the item parameters and the elements of the correlation matrix. As the marginal maximum likelihood estimator is a standard maximum likelihood estimator on basis of the marginal distribution, it has the same properties with respect to consistency, bias and its asymptotic distribution (Berger et al., 1999).

## 5 Simulation study

We conducted two simulation studies in order to investigate whether and under what conditions the model parameters and the latent traits can be estimated well. The first simulation study addressed the recovery of the item parameters, the second simulation study the recovery of the latent traits. Due to restrictions of space, we only give a limited overview of the results; see Section S2 of the Supplementary Material for further results on parameter recovery and Section S3 of the Supplementary Material for further results on trait estimation.

### 5.1 Parameter recovery

In the simulation study on parameter recovery, we generated data according to the proposed models for a test of 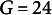 items. For Models A1–A3, the item parameter values were set to 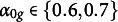 , 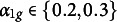 , 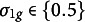 , 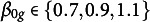 , 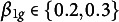 , and 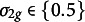 . For Models B1–B2, the item parameter values were set to 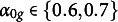 , 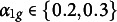 , 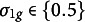 , 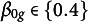 , 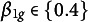 , 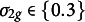 , 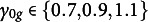 , 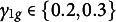 , and 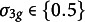 . The guessing probability was set to 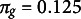 in Models A2, A3, and B2. By fully crossing the item parameters, we determined the values for 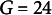 items. The values of the item parameters were within the range of values we got for the items of an intelligence test. Rather than using real items, we created synthetic ones in order to systematically cross high and low parameter values. The thresholds 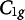 , 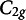 , and 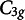 were set to 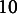 and considered as known. Fixing the thresholds to a specific value is necessary in order to identify the model. The latent traits were assumed to be multivariate normally distributed with expected values of zero and variances of one. The correlation coefficients were set to 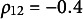 in Models A1–A3 and to 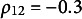 , 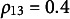 , 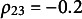 in Models B1–B3. We assumed correlated latent traits as there is evidence that capability and persistence are correlated (Zhang et al., 2024).

We generated simulation samples for all models. We simulated 250 data sets for a sample size of 250 test takers and 250 data sets for a sample size of 1,000 test takers. A description of how the data sets were generated can be found in Section S2 of the Supplementary Material. In the simulation samples, the average response times were between 3 and 4 time units and the solution frequencies between 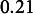 and 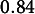 in the items. The correlation of the response times ranged from 0.04 to 0.32, the correlation of the responses from 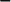 0.06 to 0.18 and the correlation of the responses and the response times from 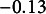 to 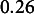 across the items and the different models. Such values should be representative for a typical achievement test.

Having generated the data sets, we estimated the item parameters with marginal maximum likelihood estimation. Details on the implementation can be found in Section S2 of the Supplementary Material. Results on parameter recovery are summarized in Table 2 for Models A2 and A3 and in Table 3 for Models B2 and B3. Results for Models A1 and B1 are not reported here, but can be found in Section S2 of the Supplementary Material (Tables S2.1–S2.4). The results were very similar and we decided to skip them in order to save space. In Tables 2 and 3, we report the true value (TV), the average estimate (M) and the standard error of estimation (SE). We also report the coverage frequency (CI) of confidence intervals for a confidence level of 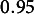 ( 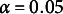 ). In both tables, the results have been averaged over parameters with the same value.

**Table 2:** True value (TV), average estimate (M), standard error of estimation (SE), and coverage frequency (CI) of confidence intervals with confidence level C=0.95 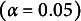 of the item parameters of the linear ballistic accumulator Model A for different samples sizes and variants of the model

	*N*=250								*N*=1,000							
	Model A2				Model A3				Model A2				Model A3			
Par	TV	M	SE	CI	TV	M	SE	CI	TV	M	SE	CI	TV	M	SE	CI
Accumulator representing progress																
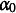	0.60	0.59	0.08	0.93	0.60	0.60	0.08	0.92	0.60	0.60	0.04	0.95	0.60	0.60	0.04	0.94
	0.70	0.69	0.07	0.94	0.70	0.70	0.08	0.93	0.70	0.70	0.03	0.96	0.70	0.70	0.04	0.95
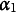	0.20	0.20	0.06	0.94	0.20	0.20	0.06	0.94	0.20	0.20	0.03	0.95	0.20	0.20	0.03	0.91
	0.30	0.30	0.07	0.94	0.30	0.30	0.07	0.93	0.30	0.30	0.03	0.95	0.30	0.30	0.03	0.91
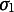	0.50	0.49	0.05	0.94	0.50	0.49	0.06	0.94	0.50	0.50	0.02	0.95	0.50	0.50	0.03	0.97
Accumulator representing disengagement																
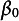	0.70	0.70	0.05	0.93	0.70	0.70	0.07	0.95	0.70	0.70	0.02	0.95	0.70	0.70	0.03	0.94
	0.90	0.90	0.05	0.93	0.90	0.90	0.06	0.93	0.90	0.90	0.02	0.94	0.90	0.90	0.03	0.95
	1.10	1.10	0.05	0.93	1.10	1.10	0.05	0.93	1.10	1.10	0.02	0.94	1.10	1.10	0.03	0.96
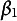	0.20	0.20	0.04	0.95	0.20	0.20	0.05	0.94	0.20	0.20	0.02	0.95	0.20	0.20	0.02	0.96
	0.30	0.30	0.04	0.95	0.30	0.30	0.05	0.95	0.30	0.30	0.02	0.95	0.30	0.30	0.03	0.95
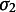	0.50	0.50	0.03	0.94	0.50	0.50	0.04	0.94	0.50	0.50	0.02	0.95	0.50	0.50	0.02	0.93
General model parameters																
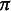	0.12	0.13	0.04	0.84	0.12	0.12	0.08	0.84	0.12	0.12	0.02	0.97	0.12	0.12	0.04	0.88
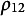	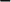 0.40	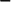 0.39	0.11	0.87	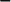 0.40	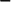 0.42	0.10	0.94	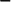 0.40	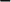 0.40	0.04	0.96	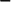 0.40	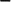 0.41	0.05	0.94

*Note:* Results for parameters have been averaged over the items of with the same parameter values; for an overview of the different models, see Table 1.

**Table 3:** True value (TV), average estimate (M), standard error of estimation (SE) and coverage frequency (CI) of confidence intervals with confidence level C=0.95 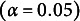 of the item parameters of the linear ballistic accumulator Model B for different samples sizes and variants of the model

	*N*=250								*N*=1,000							
	Model B2				Model B3				Model B2				Model B3			
Par	TV	M	SE	CI	TV	M	SE	CI	TV	M	SE	CI	TV	M	SE	CI
Accumulator representing progress																
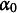	0.60	0.59	0.07	0.95	0.60	0.59	0.10	0.90	0.60	0.60	0.04	0.92	0.60	0.60	0.06	0.85
	0.70	0.69	0.06	0.96	0.70	0.69	0.09	0.91	0.70	0.70	0.03	0.93	0.70	0.70	0.05	0.86
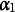	0.20	0.20	0.05	0.95	0.20	0.20	0.06	0.93	0.20	0.20	0.03	0.95	0.20	0.20	0.03	0.94
	0.30	0.30	0.06	0.96	0.30	0.30	0.06	0.94	0.30	0.30	0.03	0.95	0.30	0.30	0.03	0.94
Accumulator representing disengagement																
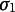	0.50	0.50	0.05	0.95	0.50	0.49	0.06	0.91	0.50	0.50	0.02	0.94	0.50	0.49	0.03	0.89
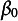	0.40	0.36	0.26	0.85	0.40	0.29	0.39	0.78	0.40	0.36	0.17	0.88	0.40	0.32	0.25	0.81
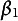	0.40	0.43	0.16	0.82	0.40	0.46	0.21	0.74	0.40	0.42	0.10	0.85	0.40	0.44	0.13	0.76
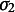	0.30	0.27	0.09	0.87	0.30	0.26	0.12	0.78	0.30	0.29	0.08	0.83	0.30	0.28	0.10	0.73
Accumulator representing misinformation																
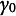	0.70	0.67	0.14	0.89	0.70	0.69	0.09	0.89	0.70	0.70	0.07	0.87	0.70	0.71	0.05	0.84
	0.90	0.87	0.11	0.93	0.90	0.89	0.08	0.91	0.90	0.90	0.05	0.88	0.90	0.90	0.04	0.86
	1.10	1.08	0.07	0.95	1.10	1.09	0.07	0.93	1.10	1.10	0.04	0.91	1.10	1.10	0.04	0.89
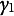	0.20	0.21	0.06	0.95	0.20	0.21	0.05	0.94	0.20	0.20	0.03	0.95	0.20	0.20	0.02	0.94
	0.30	0.31	0.07	0.94	0.30	0.30	0.05	0.95	0.30	0.30	0.03	0.94	0.30	0.30	0.03	0.94
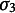	0.50	0.50	0.06	0.92	0.50	0.50	0.05	0.90	0.50	0.50	0.03	0.89	0.50	0.50	0.03	0.88
General model parameters																
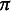	0.12	0.14	0.05	1.00	–	–	–	–	0.12	0.12	0.04	0.91	–	–	–	–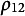	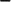 0.30	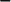 0.22	0.18	0.52	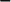 0.30	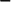 0.21	0.18	0.26	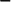 0.30	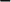 0.28	0.10	0.78	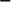 0.30	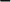 0.27	0.11	0.44
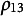	0.40	0.38	0.09	0.87	0.40	0.42	0.09	0.89	0.40	0.40	0.04	0.93	0.40	0.41	0.04	0.91
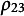	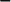 0.20	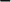 0.12	0.17	0.66	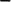 0.20	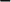 0.11	0.17	0.40	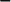 0.20	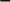 0.17	0.10	0.81	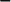 0.20	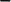 0.16	0.09	0.62

*Note:* Results for parameters have been averaged over the items of with the same parameter values; for an overview of the different models, see Table 1.

In the variants of Model A, the marginal maximum likelihood estimator performs well. The parameter estimates are virtually unbiased, irrespective of the sample size. In the condition with 1,000 subjects, the coverage frequencies of the confidence intervals are close to the intended level of 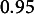 . The only exceptions are the coverage frequencies for 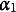 and 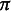 in Model A3 that are slightly too low. In the conditions with 250 subjects, the coverage frequencies are slightly too low in all parameters. In the variants of Model B, the marginal maximum likelihood estimator performs less well. Parameters of the second accumulator are biased. The coverage frequencies of the confidence intervals also fall below the intended level of 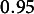 . This was partly due to the bias of the estimator, inaccurate estimates of its standard errors and deviations from the normal distribution. All this was caused by a small number of data sets where the estimates were far off the TV.

In general, the simulation study suggests that the item parameters can be estimated well with samples of at least 1000 test takers. However, in some cases, there seem to be convergence issues. In practice, it is advisable to check for problems that are indicated by extreme parameter estimates or large standard errors of estimation. Model simplifications like restricting model parameters to the same value might help in this case.

### 5.2 Recovery of latent traits

In a second simulation study, we investigated to what accuracy the latent traits of test takers can be inferred from their response and response time patterns. We defined fictitious test takers by fully crossing fixed levels of the traits ( 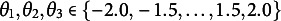 ). We used fixed trait levels in order to study whether the maximum likelihood estimator is conditionally unbiased. For each test taker, we generated 250 (Model A) or 50 (Model B) response and response time patterns for a test of 24 items. Responses and response time patterns were generated as in the simulation study on parameter recovery. We then estimated the latent traits from the response and response time patterns by maximum likelihood estimation and determined Wald-type confidence intervals for a confidence level of 0.95 ( 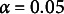 ); for more details on the implementation see Section S3 of the Supplementary Material. We repeated the simulation study for a test of 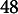 items by simply doubling the test. A detailed description of the results (e.g., average estimate, median estimate, standard error of estimation, coverage frequencies of the confidence intervals) can be found in Section S3 of the Supplementary Material. Here, we only summarize the general findings in order to save space.

In all variants of Model A, the coverage frequencies of the confidence intervals were near the intended level of 0.95. The trait estimates were virtually unbiased in Model A1 and Model A3. In Model A2, there was a small bias in low levels of capability. The standard error of estimation was generally higher in Model A2 than in Model A1 or Model A3. It was larger in low levels of the traits than in high levels. In the variants of Model B, the recovery of the traits was generally worse than in the variants of Model A. In all variants of Model B, the coverage frequencies of the confidence intervals were near the intended level of 0.95 for capability and error-proness. For persistence, the coverage frequencies were between 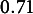 and 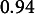 and thus below the intended level of 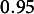 . Estimates of capability had a negligible or small bias in all models. The estimates of error-proneness were biased in low levels of the trait, although the median of the estimates was close to the TV. Average as well as median estimates of persistence deviated from the TVs in all models in case persistence was high. The standard error of estimation depended on the model and the level of the trait to be estimated. Standard errors were higher in Model B2 than in Model B1 and Model B3. Low values of capability and error-proness and high values of persistence were generally estimated with little precision.

In summary, whether trait levels can be estimated well depends on the level of the trait and on the model. Estimation is generally better in versions of Model A than in versions of Model B. Low values of capability and error-proness as well as high values of persistence are estimated with a high standard error of estimation. This is due to the race process. Whenever one accumulator dominates the others, there is little direct information about the other accumulators. All what is known is that the other accumulators were higher. This has implications for psychological assessment. It implies that in test takers with high persistence, one cannot estimate persistence well, but capability. On the other hand, in test takers with low persistence, one can estimate persistence well, but not capability. This indicates the need for an adaptive test where the time demand of an item is adjusted to the typical time investment of an individual. This, however, has limits as one cannot infer the capability of a test taker in case items are not processed at all. Adaptive tests, however, are only needed in diagnostic assessment. In large scale assessment, one typically is not interested in single test takers, but in aggregates. This does not require precise estimates on the individual level.

## 6 Empirical example

We applied the model to data from a matrix reasoning test collected by Myszkowski et al. (2022). The data consisted of the responses and response times of 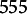 test takers on a progressive matrix reasoning test. The test comprised 17 items that were generated with the IMak R package (Blum & Holling, 2018). Each item consisted of a matrix of figures. Test takers had to deduce the rule the figures were created with. In each item, eight response options were given, one of which was the correct response. Test takers had to indicate the correct response. Responses were scored as either correct or incorrect. Data were collected online in a low-stakes setting. The data set as well as further test information are available in the repository of the original study at https://osf.io/uge2w. We chose the data set for our empirical analysis as it was collected in a low-stakes setting, such that individual differences in test taking effort are to be expected.

Before analyzing the data, we removed outliers. Some test takers had very long response times that may indicate interruptions caused by pausing or distraction. In online assessments, this cannot be avoided. As an outlier, we defined a response time that was more than 2.5 interquartile ranges above the upper quartile. We did not remove unusually short response times as we consider these as regular data. Removing outliers reduced the sample size from 555 to 543. Descriptive statistics describing the distribution of the responses and the response times in the items of the test are given in Table 4.

**Table 4:** Average solution probability ( 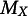 ) and range over items, average response time ( 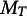 ) and range over items as well as standard deviation of response time ( 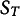 ) and range over items in the IMak test

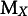	Range 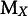	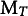	Range 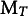	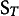	Range 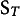
0.50	0.22–0.80	11.10	4.94–15.77	8.81	2.79–16.31

**Figure 2:**
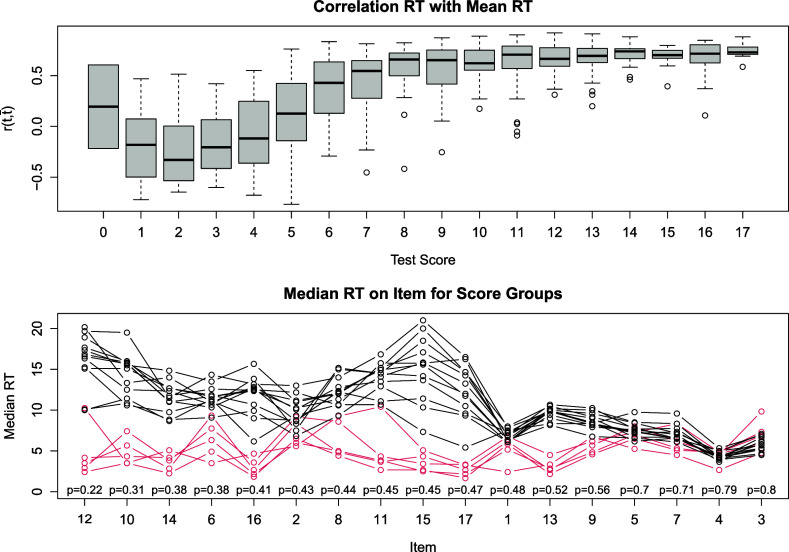
Box-plot of the correlations of the test takers’ response times with the time demand of an item given for the different score groups (upper plot) and average time on task on the items for different score groups (lower plot). *Note*: The solution probability of an item is indicated by *p*. Groups with a score of 0–4 are highlighted.

We first evaluated the pacing of the test takers. We correlated the response times of each test taker with the average time demand of an item. This resulted in 543 coefficients of correlations. A high correlation implies that a response time pattern is regular as the test taker spends more time on the more time demanding items. Figure 2, upper plot shows the distributions of the coefficients of correlations for the different score groups; note that the test score ranges from 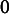 – 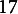 . The plot suggests that test takers with a low test score behave unusually as there is no relation between their pacing and the time demand of the items. This was corroborated by an inspection of the average time, the test takers spent on the item. Figure 2, lower plot, visualizes the average response times of the test takers in a score group for the items of the test. Each line connects the average response times of one score group over the items. Items are sorted according to their difficulty in decreasing order. Score groups with scores of 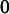 to 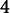 are highlighted in red. In most score groups, the average response times increase with the difficulty of the item. In low score groups, however, the response times do not to depend on the item difficulty. The figure also suggests that the item difficulty has the strongest influence on the response time. Item position effects appear negligible.

We fit all models considered in the manuscript to the IMak data by marginal maximum likelihood estimation. All models were implemented in two versions. In the first version, we assumed that all traits are multivariate normally distributed. However, given that the descriptive results suggested a high proportion of test takers with low persistence, we also implemented a version where 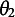 was distributed according to a mixture of two normal distributions. This more flexible distribution allows for an imbalance toward low levels of persistence that cannot be represented by the symmetric normal distribution. We did not use mixtures for the other traits as cognitive traits are supposed to be normally distributed and there was no evidence in the data against this assumption. We denote the versions with multivariate normally distributed latent traits as Models A1N to B3N and the versions with the mixture distribution as Models A1M to B3M. For sake of comparison, we also fit the hierarchical model of van der Linden (2007) to the data. The model was also implemented in two versions, using the multivariate normal distribution or a mixture of two multivariate normal distributions for the latent traits. For the hierarchical model, we used mixtures for both traits as the model does not allow for a separation between capability and persistence.

Information on relative model fit is given in Table 5 where we report the values of the marginal log-likelihood function at the parameter estimates (LL), the difference of the marginal maximum likelihood function of the respective model to the marginal maximum likelihood of the model with the highest marginal log-likelihood ( 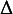 LL), the values of the Akaike Information Criterion (AIC) and the Bayesian Information Criterion (BIC) (Burnham & Anderson, 2013) as well as the difference of the respective information criterion of the current model to the information criterion of the best fitting model ( 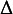 AIC and 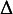 BIC).

**Table 5:** Value of the marginal log-likelihood function (LL), number of parameters (NP), difference of marginal log-likelihood function ( 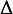 LL), AIC-index (AIC), difference of AIC-index ( 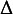 AIC), BIC-index and difference of BIC-index ( 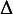 BIC) for the six accumulator models (A1–B3) and the hierarchical model (VLM) for the version with the normal distribution or the mixture distribution

Model		Version	LL	NP	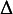 LL	AIC	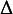 AIC	BIC	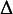 BIC
Model A	A1	N	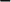 31,878.16	103	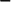 1,142.28	63,962.32	2,170.57	64,404.92	1,925.63
		M	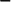 31,824.11	106	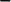 1,088.24	63,860.23	2,068.48	64,315.72	1,836.43
	A2	N	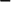 31,361.95	104	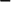 626.08	62,931.91	1,140.16	63,378.81	899.52
		M	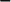 31,285.13	107	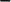 549.25	62,784.26	992.51	63,244.05	764.76
	A3	N	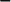 31,744.42	104	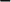 1,008.55	63,696.85	1,905.10	64,143.75	1,664.46
		M	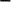 31,679.94	107	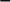 944.07	63,573.89	1,782.14	64,033.68	1,554.39
Model B	B1	N	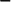 31,151.58	156	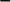 415.70	62,615.16	823.41	63,285.51	806.22
		M	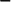 31,130.05	159	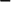 394.18	62,578.10	786.36	63,261.35	782.06
	B2	N	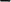 30,758.38	157	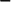 22.50	61,830.76	39.01	62,505.40	26.12
		M	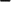 30,735.87	160	0.00	61,791.75	0.00	62,479.29	0.00
	B3	N	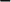 30,809.16	156	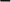 73.28	61,930.32	138.57	62,600.67	121.38
		M	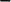 30,806.13	159	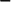 70.26	61,930.26	138.51	62,613.50	134.21
VLM	–	N	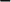 32,125.53	87	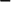 1,389.65	64,425.06	2,633.31	64,798.91	2,319.62
		M	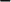 32,008.09	92	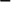 1,272.22	64,200.18	2,408.43	64,595.52	2,116.23

*Note:* Differences are with respect to the best fitting Model with lowest AIC. Model Variants are indicated as in Table 1. N denotes the normal distribution of the latent traits, M denotes the mixture distribution.

The versions of the hierarchical model of van der Linden (2007) had the worst fit. This was to be expected as already the response times were not log-normally distributed. The versions of Model A with just two accumulators were all inferior to the versions of Model B with three accumulators. This suggests that test takers produce incorrect responses actively. This could be due to the scoring of the test. In the IMak test, the more difficult items are generated by several rules. In case some, but not all rules are deduced, an incorrect response option is selected after a regular response process. In the model versions with three accumulators, the versions of model B2 and B3 that allow for guessing fit better than the versions of Model B1 without guessing. This is unexpected as in low-stakes tests, there is little to gain from guessing. Test takers, however, could not skip items so that test takers always had to choose a response option. The fit of the versions of Model B2 with random guessing was better than the fit of the versions of Model B3 with informed guessing. This could indicate that test takers who disengage preliminary are not motivated to increase their test score by strategic guessing. This is in-line with the observation that the estimated guessing probability that was around 0.18 in Model B2N and B2M, which is only slightly higher than the probability expected by chance. However, it could also be the case that informed guessing does not occur in the way it was implemented in Model B3, so that the versions of Model B3 fit worse than the potentially equally misspecificed versions of Model B2. Of the Models B2, Model B2M with the mixture distribution fit best. Normal mixtures may either be interpreted in terms of distinct classes, where each mixture component represents a qualitative different group of test takers, or as a flexible tool to generate skewed or bimodal densities without any further claim. In the present case, the mixture distribution was not bimodal, but simply had more mass in the left tail. This suggests that there are not two clearly separated classes of test takers with different level of persistence, but simply a slight imbalance toward low levels of persistence. The correlations between the latent traits were 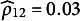 , 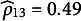 and 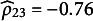 . Hence, there was no correlation between capability and persistence. There was, however, a negative correlation between persistence and the proneness toward an incorrect solution. This suggests that the tendency to give up is also related to respond incorrectly. The value of the item parameters of Model B2M with the mixture distribution as well as information on absolute model fit is given in Section S4 of the Supplementary Material.

**Figure 3:**
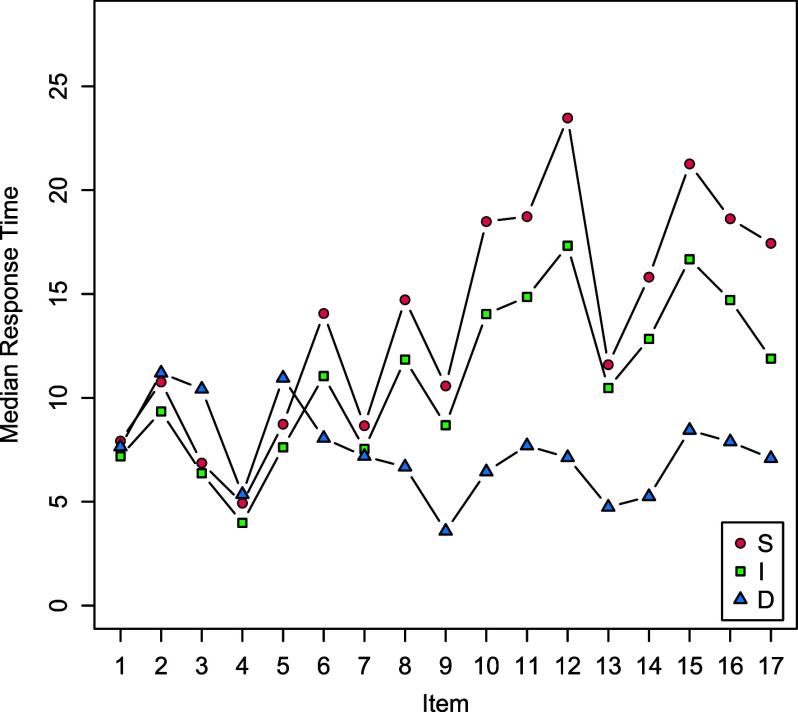
Median response times on the items that were generated by the win of the accumulator representing progress (S), disengagement (D) or misinformation (I) as implied by Model B2M and the estimated item parameters.

We analyzed the implications of Model B2M for the test taking process. Figure 3 visualizes the median response times of the winning accumulator on the items that are implied by Model B2M and the estimated item parameters. The median response times are short when the response process is interrupted (small triangular) and do not change much over the test. This corresponds to the observation that a subgroup of test takers does not increase the processing time in harder items; see Figure 2. The median response times that are generated when the progress accumulator (small dot) or the misinformation accumulator (small square) wins, are longer and increase as a function of the item difficulty; note that the median response times generated by the misinformation accumulator are slightly shorter than the ones that are generated by the progress accumulator. The profile of the median response times, however, is very similar. This suggests that the response process underlying correct and incorrect responses is similar.

**Figure 4:**
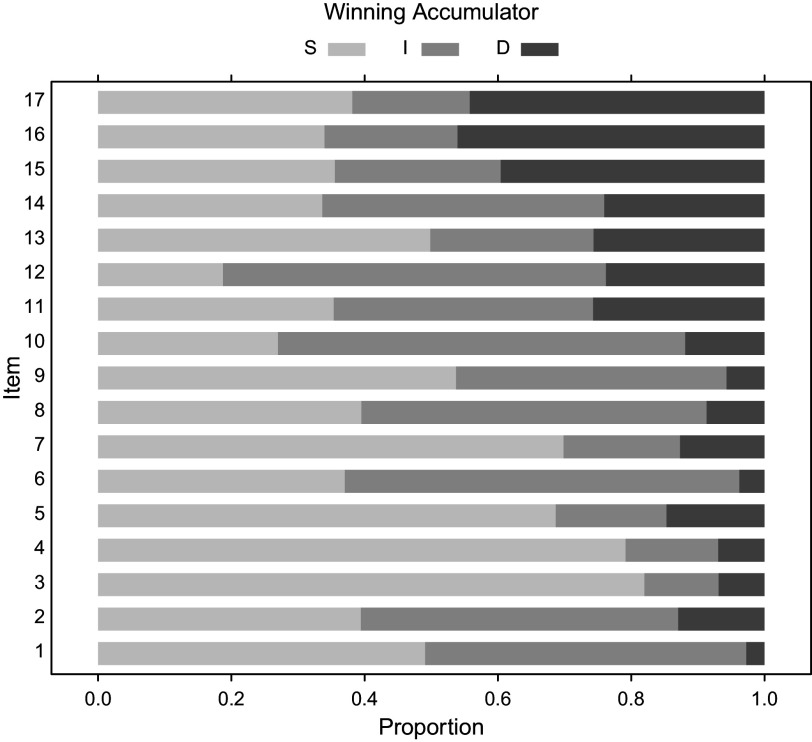
Winning probability of the accumulators representing progress (S), disengagement (D) or misinformation (I) as a function of the item position as implied by Model B2M and the estimated item parameters.

Even though the model assumes a constant level of persistence 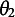 , this does not mean that the response mode of the test takers is constant throughout the test. This is illustrated in Figure 4 where the implied winning probabilities of the three accumulators are visualized for the items. The probability that the response process is interrupted preliminary increases systematically over the test. This results directly from the constant level of persistence and the increasing time demand of the more difficult items at the end of the test.

**Figure 5:**
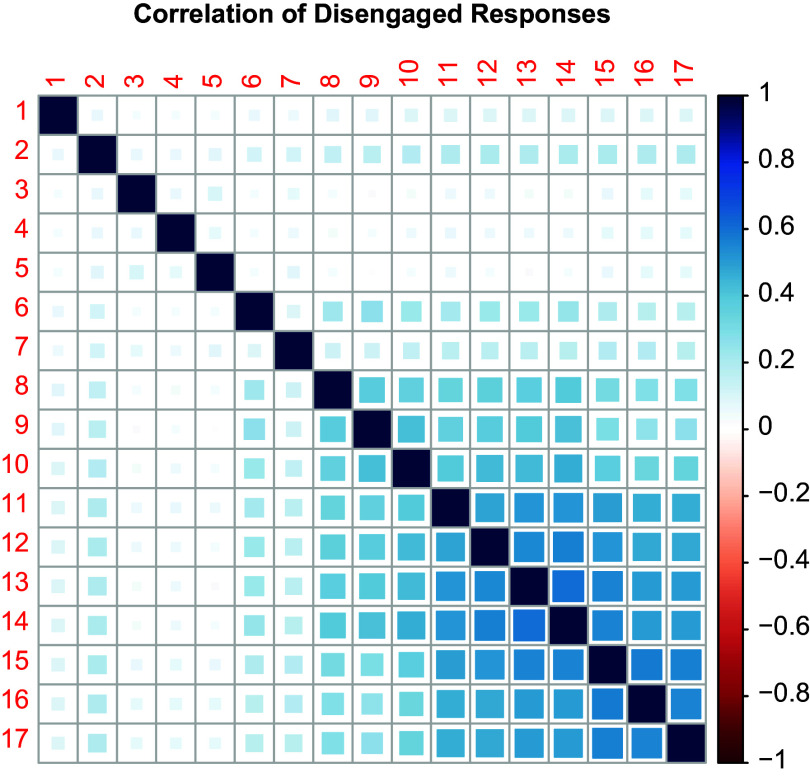
Correlations between the wins of the disengagement accumulator in the different items of the test as implied by Model B2M and the estimated item parameters.

We finally analyzed the association of the tendency to interrupt the response process in the single items; note that the accumulation of disengagement in the different items is associated due to the common influence of the persistence trait on the disengagement accumulators. The strength of the association, however, is determined by the discrimination coefficient 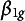 and the standard deviation of the residual 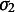 . In order to determine the strength of the association, we coded an implied win of the disengagement accumulator as one and an implied loss as zero in each item. Then we determined the correlations between the binary random variates. The correlations are visualized in Figure 5. Although the correlations also depend on the marginal probabilities, the findings suggest that the association is stronger in the last items. This is not unrealistic and might account for the fact that test takers disengage systematically when items become more demanding (and more time intensive).

## 7 Discussion

The performance in tests depends on the capability of a test taker, but also on his motivation to apply it. One important aspect of test taking motivation is the time, a test taker is willing to spend on the items. Test takers that interrupt the response process preliminary, do not reach their maximal performance level (Thurstone, 1937). In this article, we have proposed a series of models that relate the test performance to traits representing the information processing capacity of a test taker and his persistence. Core of the models is the assumption of a race between the solution process and a process of disengagement. The models differ with respect to the number of accumulators and the response in case of disengagement. In Models A1–A3, we assume two accumulators. The first accumulator acts as a kind of progress bar that reflects how near a test taker is to the solution. The second accumulator reflects the accumulating tendency to interrupt the response process. Both accumulators have different roles in the response process as the first triggers a correct response while the second triggers an incorrect response or a guessing process that is either random or informed. In Models A1–A3, incorrect responses can only occur due to lack of persistence. As such, these models are specifically suited for tests with free response format or for tests where incorrectness of a generated response is recognizable. This is the case with poorly constructed distractors. Models B1–B3 consist of three accumulators. Two accumulators reflect progress and misinformation while one accumulator represents the tendency to disengage. The progress accumulator triggers a correct response, the misinformation accumulator an incorrect response and the disengagement accumulator either triggers an incorrect response or a guessing process. Hence, we assume that incorrect responses can be generated actively, by following a wrong solution path. This is different to simply not knowing the solution. As such, these models are specifically suited for tests, in which false conclusions can be made or problems can be approached wrongly. This requires that response options in single-choice tests tap typical errors.

The models have several applications. The models provide a measure of the test taker’s capability that is purified from disengagement. In doing so, the proposed models improve upon mixture models for disengagement (e.g., Liu et al., 2019) or effort moderated item response models (e.g., Wise & DeMars, 2006) that simply classify responses as engaged or disengaged and explicitly or implicitly discard the disengaged responses when estimating the traits. This makes the model an attractive candidate for the evaluation of group performance like in the PISA study. It is also useful for psychological assessment, although the model cannot provide precise estimates of capability when persistence is very low. Apart from serving as a measurement model, the models can be used for an investigation of the response process. This was demonstrated in the empirical example where we analyzed data from a matrix reasoning test. Here, we could demonstrate that our model explains the occurrence of guesses and incorrect responses and their interrelation over time as an interplay of the time that is dedicated to a task and the time that is required to respond. We, however, failed to provide evidence for informed guessing. This might be due to the fact that test takers with very low levels of persistence are also not motivated to engage in an informed guess; or to the fact that informed guessing is difficult when a large number of distractors are presented and does not occur in the form it was implemented in the models. Whether the superiority of the models with random guessing over the models with informed guessing is a peculiarity of our data set or holds in general is a question that should be investigated in the future.

Our model is an effort to account for motivational aspects of test taking. It naturally has limitations that should be addressed in future research. First of all, the interpretation of the model in terms of persistence is not totally warranted. From a mathematical perspective, there are just accumulators that generate correct or incorrect responses or trigger a guess. This is most problematic in Model A1 and Model B1. The interpretation of accumulators in terms of disengagement is thus an interpretation that requires further evidence, e.g., predictions that imply disengagement or a relation to an external measure of test-taking motivation. Low persistence is also not necessarily a sign of low motivation, but could also be the result of meta-cognition (Cheyette & Piantadosi, 2024), fatigue or test taking strategies. A second limitation is the fact that our model is static as we consider fixed latent traits that do not change throughout the test. This is in-line with the standard approach in item response modeling. The assumption of constant trait levels is not that severe as it appears as general trends can be absorbed in the item parameters. Nevertheless, there is evidence for individual changes throughout the test (e.g., Schweizer et al., 2020; Ulitzsch et al., 2022; Weirich et al., 2017). Modeling individual changes requires a combination of the race model with growth curve models. This is a topic of future research. A third limitation is the assumption that test takers will always give a response. Test takers that quit working may also skip the item entirely. This results in item omissions. As item omissions indicate a lack of persistence, they can legitimately be scored as wrong responses in models A1 and B1. This does not hold for models A2/A3 and models B2/B3 where there is no one-to-one correspondence between quitting and a wrong response. Incorporating missing responses into the models, however, is not trivial. As in models A3 and B3, the given response under quitting depends on the accumulated knowledge, the accumulated knowledge should also influence the decision between omission and guessing. Such an extension of the proposed models to omitted responses is a topic for future research. At the moment, it might be best to prevent omissions by not allowing the test takers to skip items. And finally, the assumption of a race between a disengagement process and a solution process is not the only possible approach. First of all, the decision to disengage might be a direct function of the progress a test taker is actually making. This requires assumptions about the meta-cognitions of a test taker that monitor the process of test taking. Second, there are alternative process models that could be extended, like the diffusion model with collapsing boundaries.

## Supporting information

Ranger et al. supplementary material Ranger et al. supplementary material

## Data Availability

The IMak data are available via OSF at https://osf.io/uge2w.

## Footnotes

1 There are more approaches to account for persistence, like the identification of non-effortful responses (e.g., Liu et al., 2019; Wise & DeMars, 2006), models that use response time as a predictor of response accuracy (e.g., Bolsinova et al., 2011; van Breukelen & Roskam, 1991; Wang & Hanson, 2005) or mixture models solely based on the responses (e.g., Bolt et al., 2002; Yamamoto & Everson, 1995). We do not review them in detail as they are not relevant for the manuscript.

2 Fixing the threshold over test takers forces the accumulators to the same scale. This does not affect the interpretation of the item parameters in case the scale is item specific. Such an assumption is reasonable for the progress and misinformation accumulator that represent critical knowledge levels that are not subject to deliberate control. Individual variations in the scale, however, might occur in the disengagement accumulator. Random variations in the disengagement threshold, should they exist, are transferred directly to random fluctuations of the disengagement accumulator when the threshold is fixed. It is thus not possible to distinguish individual differences in the threshold from individual differences in the drift rate.

## Supplementary material

The supplementary material for this article can be found at https://doi.org/10.1017/psy.2025.10026.
