# Supplementary material for: Accounting for Persistence in Tests with Linear Ballistic Accumulator Models
Source: Psychometrika. 2025 Jun 16;90(3):1111–35. doi: 10.1017/psy.2025.10026 (PMC12483707; doi:10.1017/psy.2025.10026)
Supplement: Ranger et al. supplementary material [file S0033312325100264sup001.pdf]

Supplement S1: Subdensity Functions of the Proposed Race Models

The following section lists the subdensities of the response time models in Table 1. For ease of notation, the subscript denoting the item has been omitted. Function $\phi(x)$ denotes the density function of the standard normal distribution. Function $\mathrm{S}(x)$ denotes the survival function of the standard normal distribution. Linear predictors are denoted by $a(\theta_1) = \alpha_0 + \alpha_1\theta_1$, $b(\theta_2) = \beta_0 - \beta_1\theta_2$ and $c(\theta_3) = \gamma_0 + \gamma_1\theta_3$. The thresholds are all set to $C_1 = C_2 = C_3 = 10$.

Model A1

$$\mathrm{f}\Big(t, x = 1|\theta_1, \theta_2\Big) = \frac{1}{\sigma_1} \cdot \frac{1}{t} \cdot \phi\left(\frac{\log(t) - \log(10) + a(\theta_1)}{\sigma_1}\right) \cdot \mathrm{S}\left(\frac{\log(t) - \log(10) + b(\theta_2)}{\sigma_2}\right)$$

$$\mathrm{f}\Big(t, x = 0|\theta_1, \theta_2\Big) = \frac{1}{\sigma_2} \cdot \frac{1}{t} \cdot \phi\left(\frac{\log(t) - \log(10) + b(\theta_2)}{\sigma_2}\right) \cdot \mathrm{S}\left(\frac{\log(t) - \log(10) + a(\theta_1)}{\sigma_1}\right)$$

Model A2

$$\begin{aligned}\mathrm{f}\Big(t, x = 1|\theta_1, \theta_2\Big) =& \frac{1}{\sigma_1} \cdot \frac{1}{t} \cdot \phi\left(\frac{\log(t) - \log(10) + a(\theta_1)}{\sigma_1}\right) \cdot \mathrm{S}\left(\frac{\log(t) - \log(10) + b(\theta_2)}{\sigma_2}\right) \\ &+ \pi \cdot \frac{1}{\sigma_2} \cdot \frac{1}{t} \cdot \phi\left(\frac{\log(t) - \log(10) + b(\theta_2)}{\sigma_2}\right) \cdot \mathrm{S}\left(\frac{\log(t) - \log(10) + a(\theta_1)}{\sigma_1}\right)\end{aligned}$$

$$\mathrm{f}\Big(t, x = 0|\theta_1, \theta_2\Big) = (1 - \pi) \cdot \frac{1}{\sigma_2} \cdot \frac{1}{t} \cdot \phi\left(\frac{\log(t) - \log(10) + b(\theta_2)}{\sigma_2}\right) \cdot \mathrm{S}\left(\frac{\log(t) - \log(10) + a(\theta_1)}{\sigma_1}\right)$$

Model A3

$$\mathrm{f}\Big(t, x = 1|\theta_1, \theta_2\Big) = \frac{1}{\sigma_1} \cdot \frac{1}{t} \cdot \phi\left(\frac{\log(t) - \log(10) + a(\theta_1)}{\sigma_1}\right) \cdot \mathrm{S}\left(\frac{\log(t) - \log(10) + b(\theta_2)}{\sigma_2}\right)$$
$$+ \frac{1}{\sigma_2} \cdot \frac{1}{t} \cdot \phi\left(\frac{\log(t) - \log(10) + b_g(\theta_2)}{\sigma_2}\right) \cdot \left[\pi \cdot \mathrm{S}\left(\frac{\log(t) - \log(10) + a(\theta_1)}{\sigma_1}\right)\right.$$
$$+ (1-\pi) \cdot t \cdot \mathrm{S}\left(\frac{\log(t) - \log(10) + a(\theta_1) + \sigma_1^2}{\sigma_1}\right)$$
$$\left.\cdot \exp\left(\frac{1}{2} \cdot \frac{1}{\sigma_1^2} \cdot \left[\Big(\log(10) - a(\theta_1) - \sigma_1^2\Big)^2 - \Big(\log(10) - a(\theta_1)\Big)^2\right]\right)\right]$$

$$\mathrm{f}\Big(t, x = 0|\theta_1, \theta_2\Big) = \frac{1}{\sigma_2} \cdot \frac{1}{t} \cdot \phi\left(\frac{\log(t) - \log(10) + b(\theta_2)}{\sigma_2}\right) \cdot (1-\pi)$$
$$\cdot \left[\mathrm{S}\left(\frac{\log(t) - \log(10) + a(\theta_1)}{\sigma_1}\right) - t \cdot \mathrm{S}\left(\frac{\log(t) - \log(10) + a(\theta_1) + \sigma_1^2}{\sigma_1}\right)\right.$$
$$\left.\cdot \exp\left(\frac{1}{2} \cdot \frac{1}{\sigma_1^2} \cdot \left[\Big(\log(10) - a(\theta_1) - \sigma_1^2\Big)^2 - \Big(\log(10) - a(\theta_1)\Big)^2\right]\right)\right]$$

Model B1

$$\mathrm{f}\Big(t, x = 1|\theta_1, \theta_2, \theta_3\Big) = \frac{1}{\sigma_1} \cdot \frac{1}{t} \cdot \phi\left(\frac{\log(t) - \log(10) + a(\theta_1)}{\sigma_1}\right) \cdot \mathrm{S}\left(\frac{\log t - \log 10 + b(\theta_2)}{\sigma_2}\right)$$
$$\cdot \mathrm{S}\left(\frac{\log t - \log 10 + c(\theta_3)}{\sigma_3}\right)$$

$$\mathrm{f}\Big(t, x = 0|\theta_1, \theta_2, \theta_3\Big) = \frac{1}{\sigma_3} \cdot \frac{1}{t} \cdot \phi\left(\frac{\log(t) - \log(10) + c(\theta_3)}{\sigma_3}\right) \cdot \mathrm{S}\left(\frac{\log t - \log(10) + a(\theta_1)}{\sigma_1}\right)$$
$$\cdot \mathrm{S}\left(\frac{\log(t) - \log(10) + b(\theta_2)}{\sigma_2}\right) + \frac{1}{\sigma_2} \cdot \frac{1}{t} \cdot \phi\left(\frac{\log(t) - \log(10) + b(\theta_2)}{\sigma_2}\right)$$
$$\cdot \mathrm{S}\left(\frac{\log(t) - \log(10) + a(\theta_1)}{\sigma_1}\right) \cdot \mathrm{S}\left(\frac{\log(t) - \log(10) + c(\theta_3)}{\sigma_3}\right)$$

Model B2

$$\begin{aligned}\mathrm{f}\Big(t, x=1|\theta_1,\theta_2,\theta_3\Big) &= \frac{1}{\sigma_1}\cdot\frac{1}{t}\cdot\phi\left(\frac{\log(t)-\log(10)+a(\theta_1)}{\sigma_1}\right)\cdot \mathrm{S}\left(\frac{\log(t)-\log(10)+b(\theta_2)}{\sigma_2}\right)\\ &\cdot \mathrm{S}\left(\frac{\log(t)-\log(10)+c(\theta_3)}{\sigma_3}\right) + \pi\cdot\frac{1}{\sigma_2}\cdot\frac{1}{t}\cdot\phi\left(\frac{\log(t)-\log(10)+b(\theta_2)}{\sigma_2}\right)\\ &\cdot \mathrm{S}\left(\frac{\log(t)-\log(10)+a(\theta_1)}{\sigma_1}\right)\cdot \mathrm{S}\left(\frac{\log(t)-\log(10)+c(\theta_3)}{\sigma_3}\right)\end{aligned}$$

$$\begin{aligned}\mathrm{f}\Big(t, x=0|\theta_1,\theta_2,\theta_3\Big) &= \frac{1}{\sigma_3}\cdot\frac{1}{t}\cdot\phi\left(\frac{\log(t)-\log(10)+c(\theta_3)}{\sigma_3}\right)\cdot \mathrm{S}\left(\frac{\log(t)-\log(10)+a(\theta_1)}{\sigma_1}\right)\\ &\cdot \mathrm{S}\left(\frac{\log(t)-\log(10)+b(\theta_2)}{\sigma_2}\right) + (1-\pi)\cdot\frac{1}{\sigma_2}\cdot\frac{1}{t}\cdot\phi\left(\frac{\log(t)-\log(10)+b(\theta_2)}{\sigma_2}\right)\\ &\cdot \mathrm{S}\left(\frac{\log(t)-\log 10+a(\theta_1)}{\sigma_1}\right)\cdot \mathrm{S}\left(\frac{\log(t)-\log(10)+c(\theta_3)}{\sigma_3}\right)\end{aligned}$$

Model B3

$$\begin{aligned}\mathrm{f}\Big(t, x=1|\theta_1,\theta_2,\theta_3\Big) &= \frac{1}{\sigma_1}\cdot\frac{1}{t}\cdot\phi\left(\frac{\log(t)-\log(10)+a(\theta_1)}{\sigma_1}\right)\cdot \mathrm{S}\left(\frac{\log(t)-\log(10)+b(\theta_2)}{\sigma_2}\right)\\ &\cdot \mathrm{S}\left(\frac{\log(t)-\log(10)+c(\theta_3)}{\sigma_3}\right) + \frac{1}{\sigma_2}\cdot\frac{1}{t}\cdot\phi\left(\frac{\log(t)-\log(10)+b(\theta_2)}{\sigma_2}\right)\\ &\cdot\int_t^\infty \frac{1}{\sigma_1}\cdot\frac{1}{z}\cdot\phi\left(\frac{\log(z)-\log(10)+a(\theta_1)}{\sigma_1}\right)\cdot \mathrm{S}\left(\frac{\log(z)-\log(10)+c(\theta_3)}{\sigma_3}\right)\mathrm{d}z\end{aligned}$$

$$\begin{aligned}\mathrm{f}\Big(t, x=0|\theta_1,\theta_2,\theta_3\Big) &= \frac{1}{\sigma_3}\cdot\frac{1}{t}\cdot\phi\left(\frac{\log(t)-\log(10)+c(\theta_3)}{\sigma_3}\right)\cdot \mathrm{S}\left(\frac{\log(t)-\log(10)+a(\theta_1)}{\sigma_1}\right)\\ &\cdot \mathrm{S}\left(\frac{\log(t)-\log(10)+b(\theta_2)}{\sigma_2}\right) + \frac{1}{\sigma_2}\cdot\frac{1}{t}\cdot\phi\left(\frac{\log(t)-\log(10)+b(\theta_2)}{\sigma_2}\right)\\ &\cdot\int_t^\infty \frac{1}{\sigma_3}\cdot\frac{1}{z}\cdot\phi\left(\frac{\log(z)-\log(10)+c(\theta_3)}{\sigma_3}\right)\cdot \mathrm{S}\left(\frac{\log(z)-\log 10+a(\theta_1)}{\sigma_1}\right)\mathrm{d}z\end{aligned}$$

Supplement S2: Simulation Study on the Recovery of the Item Parameters

In the simulation study on parameter recovery, we generated data according to the proposed models for a test of $G = 24$ items. For Models A1–A3, the item parameter values were set to $\alpha_{0g} \in \{0.6, 0.7\}$, $\alpha_{1g} \in \{0.2, 0.3\}$, $\sigma_{1g} \in \{0.5\}$, $\beta_{0g} \in \{0.7, 0.9, 1.1\}$, $\beta_{1g} \in \{0.2, 0.3\}$ and $\sigma_{2g} \in \{0.5\}$. For Models B1–B2, the item parameter values were set to $\alpha_{0g} \in \{0.6, 0.7\}$, $\alpha_{1g} \in \{0.2, 0.3\}$, $\sigma_{1g} \in \{0.5\}$, $\beta_{0g} \in \{0.4\}$, $\beta_{1g} \in \{0.4\}$, $\sigma_{2g} \in \{0.3\}$, $\gamma_{0g} \in \{0.7, 0.9, 1.1\}$, $\gamma_{1g} \in \{0.2, 0.3\}$ and $\sigma_{3g} \in \{0.5\}$. The guessing probability was set to $\pi_g = 0.125$ in Models A2, A3 and B2. By fully crossing the item parameters, we determined the values for $G = 24$ items. The latent traits were assumed to be multivariate normally distributed with expected values of zero and variances of one. The correlation coefficients were set to $\rho_{12} = -0.4$ in Models A1–A3 and to $\rho_{12} = -0.3$, $\rho_{13} = 0.4$, $\rho_{23} = -0.2$ in Models B1–B3.

Data were generated as follows. For each fictitious test taker, we randomly drew values of the latent traits from the specified multivariate normal distribution. We then generated the drift rates according to Equation 1, Equation 3 and Equation 6. The hitting times of the accumulators were generated as $T_{Sg} = 10/\alpha_g(\theta_1)$, $T_{Dg} = 10/\beta_g(\theta_2)$ and $T_{Ig} = 10/\gamma_g(\theta_3)$. The observed response time was set to the smallest of the generated hitting times of a test taker in an item. The response was determined as follows. When the accumulator of progress was the first to hit its threshold, the observed response was always correct. When the disengagement accumulator was the first to hit its threshold, the response was an incorrect response in Model A1 and Model B1, a random guess in Model A2 and Model B2 and an informed guess in Model A3 and Model B3. Random guesses were simulated by random draws from a Bernoulli distribution with guessing probability of $\pi_g = 0.125$ in all items. Informed guesses were generated as described in the main document. In this manner, we generated data sets for the six models with 250 or 1000 test takers. Within each condition defined by a model and a sample size, we generated and analyzed 250 data sets.

For each data set, we determined the marginal maximum likelihood estimates of the model parameters. This consisted in maximizing the marginal likelihood function

given in Equation 9 over the model parameters. The integral over the distribution of the latent traits in the marginal log-likelihood function was approximated by Gauss Hermite Quadrature with 20 nodes per dimension in Model A1 to B2 and with 10 nodes per dimension in Model B3. We reduced the number of nodes in Model B3 as this model was computationally more intensive than the other models. In order to simplify the models, the estimates of the guessing probability in all item parameters were restricted to the same value ($\hat{\pi}_g = \hat{\pi}$). All other parameters were estimated freely. The correlation matrix was re-parametrized in terms of the z-transformed correlations ρ_{12}, ρ_{13} and the partial correlation $\rho_{23.1}$ in order to avoid non-positive definite correlation matrices. Variances were log-transformed and the guessing probability was parameterized in terms of its logit. The marginal log-likelihood function was optimized with the `BFGS` algorithm (Nocedal & Wright, 2006) with analytical derivatives. For all estimates, we determined confidence intervals. The standard errors of estimation were calculated with the expected information matrix. The expected information matrix was approximated by a Monte Carlo approach with 10000 simulated data vectors. The simulation study was implemented in the statistical software environment `R`.

Table S2.1 to Table S2.4 inform about parameter recovery with respect to average estimate, standard error of estimation and coverage frequencies of confidence intervals.

Table S2.1

True Value (TV), Average Estimate (M), Standard Error of Estimation (SE) and Coverage Frequency (CI) of Confidence Intervals with Confidence Level C=0.95 ($\alpha = 0.05$) *of the Item Parameters of the Linear Ballistic Accumulator Model A for Different Variants of the Model in Samples of* $N = 250$ *Test Takers.*

	Model A1				Model A2				Model A3			
Par	TV	M	SE	CI	TV	M	SE	CI	TV	M	SE	CI
α_0	0.60	0.60	0.05	0.96	0.60	0.59	0.08	0.93	0.60	0.60	0.08	0.92
	0.70	0.70	0.05	0.95	0.70	0.69	0.07	0.94	0.70	0.70	0.08	0.93
α_1	0.20	0.20	0.05	0.94	0.20	0.20	0.06	0.94	0.20	0.20	0.06	0.94
	0.30	0.30	0.05	0.95	0.30	0.30	0.07	0.94	0.30	0.30	0.07	0.93
σ_1	0.50	0.50	0.04	0.95	0.50	0.49	0.05	0.94	0.50	0.49	0.06	0.94
β_0	0.70	0.70	0.04	0.95	0.70	0.70	0.05	0.93	0.70	0.70	0.07	0.95
	0.90	0.90	0.04	0.95	0.90	0.90	0.05	0.93	0.90	0.90	0.06	0.93
	1.10	1.10	0.04	0.94	1.10	1.10	0.05	0.93	1.10	1.10	0.05	0.93
β_1	0.20	0.20	0.04	0.94	0.20	0.20	0.04	0.95	0.20	0.20	0.05	0.94
	0.30	0.30	0.04	0.95	0.30	0.30	0.04	0.95	0.30	0.30	0.05	0.95
σ_2	0.50	0.50	0.03	0.94	0.50	0.50	0.03	0.94	0.50	0.50	0.04	0.94
π	–	–	–	–	0.12	0.13	0.04	0.84	0.12	0.12	0.08	0.84
ρ_{12}	-0.40	-0.40	0.07	0.96	-0.40	-0.39	0.11	0.87	-0.40	-0.42	0.10	0.94

Note. Results for parameters have been averaged over the items of with the same parameter values; for an overview of the different models see Table 1.

Table S2.2

True Value (TV), Average Estimate (M), Standard Error of Estimation (SE) and Coverage Frequency (CI) of Confidence Intervals with Confidence Level C=0.95 ($\alpha = 0.05$) *of the Item Parameters of the Linear Ballistic Accumulator Model A for Different Variants of the Model in Samples of* $N = 1000$ *Test Takers.*

	Model A1				Model A2				Model A3			
Par	TV	M	SE	CI	TV	M	SE	CI	TV	M	SE	CI
α_0	0.60	0.60	0.03	0.95	0.60	0.60	0.04	0.95	0.60	0.60	0.04	0.94
	0.70	0.70	0.02	0.95	0.70	0.70	0.03	0.96	0.70	0.70	0.04	0.95
α_1	0.20	0.20	0.02	0.95	0.20	0.20	0.03	0.95	0.20	0.20	0.03	0.91
	0.30	0.30	0.02	0.95	0.30	0.30	0.03	0.95	0.30	0.30	0.03	0.91
σ_1	0.50	0.50	0.02	0.95	0.50	0.50	0.02	0.95	0.50	0.50	0.03	0.97
β_0	0.70	0.70	0.02	0.95	0.70	0.70	0.02	0.95	0.70	0.70	0.03	0.94
	0.90	0.90	0.02	0.95	0.90	0.90	0.02	0.94	0.90	0.90	0.03	0.95
	1.10	1.10	0.02	0.96	1.10	1.10	0.02	0.94	1.10	1.10	0.03	0.96
β_1	0.20	0.20	0.02	0.95	0.20	0.20	0.02	0.95	0.20	0.20	0.02	0.96
	0.30	0.30	0.02	0.96	0.30	0.30	0.02	0.95	0.30	0.30	0.03	0.95
σ_2	0.50	0.50	0.01	0.95	0.50	0.50	0.02	0.95	0.50	0.50	0.02	0.93
π	–	–	–	–	0.12	0.12	0.02	0.97	0.12	0.12	0.04	0.88
ρ_{12}	-0.40	-0.40	0.03	0.96	-0.40	-0.40	0.04	0.96	-0.40	-0.41	0.05	0.94

Note. Results for parameters have been averaged over the items of with the same parameter values; for an overview of the different models see Table 1.

Table S2.3

True Value (TV), Average Estimate (M), Standard Error of Estimation (SE) and Coverage Frequency (CI) of Confidence Intervals with Confidence Level C=0.95 ($\alpha = 0.05$) *of the Item Parameters of the Linear Ballistic Accumulator Model B for Different Variants of the Model in Samples of* $N = 250$ *Test Takers.*

	Model B1				Model B2				Model B3			
Par	TV	M	SE	CI	TV	M	SE	CI	TV	M	SE	CI
α_0	0.60	0.60	0.06	0.95	0.60	0.59	0.07	0.95	0.60	0.59	0.10	0.90
	0.70	0.70	0.05	0.95	0.70	0.69	0.06	0.96	0.70	0.69	0.09	0.91
α_1	0.20	0.20	0.05	0.94	0.20	0.20	0.05	0.95	0.20	0.20	0.06	0.93
	0.30	0.30	0.05	0.96	0.30	0.30	0.06	0.96	0.30	0.30	0.06	0.94
σ_1	0.50	0.50	0.04	0.95	0.50	0.50	0.05	0.95	0.50	0.49	0.06	0.91
β_0	0.40	0.37	0.24	0.86	0.40	0.36	0.26	0.85	0.40	0.29	0.39	0.78
β_1	0.40	0.42	0.15	0.84	0.40	0.43	0.16	0.82	0.40	0.46	0.21	0.74
σ_2	0.30	0.28	0.09	0.87	0.30	0.27	0.09	0.87	0.30	0.26	0.12	0.78
γ_0	0.70	0.67	0.13	0.90	0.70	0.67	0.14	0.89	0.70	0.69	0.09	0.89
	0.90	0.88	0.10	0.94	0.90	0.87	0.11	0.93	0.90	0.89	0.08	0.91
	1.10	1.09	0.07	0.94	1.10	1.08	0.07	0.95	1.10	1.09	0.07	0.93
γ_1	0.20	0.21	0.06	0.94	0.20	0.21	0.06	0.95	0.20	0.21	0.05	0.94
	0.30	0.31	0.07	0.93	0.30	0.31	0.07	0.94	0.30	0.30	0.05	0.95
σ_3	0.50	0.50	0.06	0.92	0.50	0.50	0.06	0.92	0.50	0.50	0.05	0.90
π	–	–	–	–	0.12	0.14	0.05	1.00	–	–	–	–
ρ_{12}	-0.30	-0.26	0.14	0.57	-0.30	-0.22	0.18	0.52	-0.30	-0.21	0.18	0.26
ρ_{13}	0.40	0.38	0.08	0.93	0.40	0.38	0.09	0.87	0.40	0.42	0.09	0.89
ρ_{23}	-0.20	-0.13	0.15	0.74	-0.20	-0.12	0.17	0.66	-0.20	-0.11	0.17	0.40

Note. Results for parameters have been averaged over the items of with the same parameter values; for an overview of the different models see Table 1.

Table S2.4

True Value (TV), Average Estimate (M), Standard Error of Estimation (SE) and Coverage Frequency (CI) of Confidence Intervals with Confidence Level C=0.95 ($\alpha = 0.05$) *of the Item Parameters of the Linear Ballistic Accumulator Model B for Different Variants of the Model in Samples of* $N = 1000$ *Test Takers.*

	Model B1				Model B2				Model B3			
Par	TV	M	SE	CI	TV	M	SE	CI	TV	M	SE	CI
α_0	0.60	0.60	0.03	0.95	0.60	0.60	0.04	0.92	0.60	0.60	0.06	0.85
	0.70	0.70	0.03	0.94	0.70	0.70	0.03	0.93	0.70	0.70	0.05	0.86
α_1	0.20	0.20	0.02	0.95	0.20	0.20	0.03	0.95	0.20	0.20	0.03	0.94
	0.30	0.30	0.03	0.96	0.30	0.30	0.03	0.95	0.30	0.30	0.03	0.94
σ_1	0.50	0.50	0.02	0.95	0.50	0.50	0.02	0.94	0.50	0.49	0.03	0.89
β_0	0.40	0.38	0.14	0.90	0.40	0.36	0.17	0.88	0.40	0.32	0.25	0.81
β_1	0.40	0.42	0.08	0.87	0.40	0.42	0.10	0.85	0.40	0.44	0.13	0.76
σ_2	0.30	0.29	0.06	0.87	0.30	0.29	0.08	0.83	0.30	0.28	0.10	0.73
γ_0	0.70	0.70	0.06	0.91	0.70	0.70	0.07	0.87	0.70	0.71	0.05	0.84
	0.90	0.90	0.04	0.91	0.90	0.90	0.05	0.88	0.90	0.90	0.04	0.86
	1.10	1.10	0.04	0.92	1.10	1.10	0.04	0.91	1.10	1.10	0.04	0.89
γ_1	0.20	0.20	0.03	0.95	0.20	0.20	0.03	0.95	0.20	0.20	0.02	0.94
	0.30	0.30	0.03	0.95	0.30	0.30	0.03	0.94	0.30	0.30	0.03	0.94
σ_3	0.50	0.50	0.03	0.92	0.50	0.50	0.03	0.89	0.50	0.50	0.03	0.88
π	–	–	–	–	0.12	0.12	0.04	0.91	–	–	–	–
ρ_{12}	-0.30	-0.28	0.06	0.85	-0.30	-0.28	0.10	0.78	-0.30	-0.27	0.11	0.44
ρ_{13}	0.40	0.40	0.04	0.94	0.40	0.40	0.04	0.93	0.40	0.41	0.04	0.91
ρ_{23}	-0.20	-0.16	0.09	0.82	-0.20	-0.17	0.10	0.81	-0.20	-0.16	0.09	0.62

Note. Results for parameters have been averaged over the items of with the same parameter values; for an overview of the different models see Table 1.

Supplement S3: Simulation Study on the Recovery of the Latent Traits

In a second simulation study, we investigated to what accuracy the latent traits of test takers can be inferred from their response and response time pattern. This informs about the utility of the proposed models for psychological assessment. We conducted two studies. In a first study, we assessed the bias and the coverage frequencies of confidence intervals when the traits are estimated with maximum likelihood estimation and the data generating model is used. In a second study, we assessed whether the traits can also be recovered with the hierarchical model of van der Linden (2007). In this study, we investigated whether trait estimates derived with the hierarchical model can be used as a proxy for the latent capability θ_1. The hierarchical model is simpler than the proposed race models and would probably be the first model considered for data analysis.

Study on Bias of Estimates and Coverage Frequency of Confidence Intervals

In a first study, we investigated the trait recovery for the proposed models. We generated data as follows. We defined fictitious test takers by fully crossing fixed levels of the different traits. We defined 9×9 fictitious test takers for Models A1–A3 and $9 \times 9 \times 9$ fictitious test takers for Models B1–B3 by fully crossing the nine trait levels $\{-2.0, -1.5, \ldots, 1.5, 2.0\}$ for each trait. We used fixed trait levels in order to study whether the maximum likelihood estimator is conditionally unbiased. For each test taker and model variant, we generated responses and response times for a test of 24 items. The item parameters of the test items were identical to the item parameters used in the simulation study on parameter recovery; see the main document. We then estimated the latent traits of the fictitious test takers by maximum likelihood estimation. For this purpose, we considered the distribution function of the responses and response times (Equation 8) as a function of the latent traits. For given responses and response times, we determined those trait values that maximized this function. The values of the item parameters considered as known and set to the true values. We proceeded in this way as we did not want to confound item parameter estimation and trait estimation. In addition to point estimates of the trait levels, we determined

Wald-type confidence intervals for a confidence level of 0.95 ($\alpha = 0.05$). The standard errors of estimation were determined by inverting the test information matrix. The process of data generation and trait estimation was replicated for each test taker 250 times in Model A and 50 times in Model B. This resulted in 81×250 trait estimates and confidence intervals in the variants of Model A and 729×50 trait estimates and confidence intervals in the variants of Model B per trait level.

We repeated the simulation study for a test of 48 items. We proceeded as before by defining 9×9 or $9 \times 9 \times 9$ fictitious test takers. This time, however, we generated twice as many responses and response times. In order to double the length of the test, we used each of the 24 items twice. Having generated the data, we estimated the latent traits and determined confidence intervals as before. Table S3.1 to Table S3.4 contain the average estimates, the median estimates, the standard errors of estimation and the coverage frequencies of the confidence intervals.

Study on Using the Hierarchical Model for the Estimation of Capability

In a second simulation study, we addressed the question whether the latent capability can also be recovered with the hierarchical model of van der Linden (2007). The hierarchical model is simpler than the proposed race models and might be the first choice for data analysis. For this purpose, we generated six new data sets. Data were generated as in the study on trait recovery with one exception. Instead of using fixed trait levels, we draw the latent traits from a multivariate normal distribution. This mimics the distribution of the traits in a random sample of test takers. For each model, we generated one data set containing the responses and response times for 48 items and 10000 test takers. We then estimated the latent traits with the data generating model (Model A1–B3) by maximum likelihood estimation. We also estimated a proxy of the latent capability with the hierarchical model. We proceeded as follows. We first fit a 2-PL model (Embretson & Reise, 2000) and a log-normal factor model (van der Linden, 2006) to the data sets by marginal maximum likelihood estimation. As the models are misspecified, the resulting item parameter estimates are quasi-maximum likelihood estimates (White, 1982). We then used the fitted models to estimate the effective

Table S3.1

True Value (TV), Number of Converged Replications (N), Average Estimate (M), Median Estimate (MD), Standard Error of Estimation (SE) and Coverage Frequency (CI) of Confidence Intervals with Confidence Level C=0.95 ($\alpha = 0.05$) of the Traits of the Linear Ballistic Accumulator Model A for Different Variants of the Model and a Test of 24 Items.

		Model A1					Model A2					Model A3				
TR	TV	N	M	MD	SE	CI	N	M	MD	SE	CI	N	M	MD	SE	CI
θ_1	-2.00	2074	-2.04	-1.98	0.73	0.96	2239	-2.64	-2.04	2.42	0.95	2250	-2.08	-1.99	1.05	0.95
	-1.50	2147	-1.56	-1.50	0.67	0.96	2247	-1.98	-1.55	1.99	0.96	2249	-1.55	-1.49	0.94	0.94
	-1.00	2206	-1.07	-1.00	0.67	0.95	2244	-1.34	-1.00	1.79	0.94	2246	-1.04	-0.96	0.89	0.94
	-0.50	2222	-0.58	-0.54	0.62	0.95	2250	-0.74	-0.51	1.50	0.96	2238	-0.56	-0.49	0.79	0.95
	0.00	2248	-0.08	-0.04	0.57	0.95	2249	-0.13	0.00	1.09	0.96	2230	-0.04	0.01	0.70	0.94
	0.50	2250	0.45	0.47	0.54	0.94	2250	0.37	0.46	0.92	0.96	2195	0.46	0.50	0.66	0.95
	1.00	2250	0.97	1.00	0.49	0.95	2248	0.93	1.00	0.76	0.96	2146	0.93	0.96	0.59	0.97
	1.50	2250	1.50	1.52	0.47	0.95	2245	1.46	1.48	0.51	0.96	2087	1.46	1.49	0.56	0.96
	2.00	2248	1.97	1.99	0.47	0.95	2242	1.98	2.00	0.49	0.95	1976	1.96	1.99	0.52	0.96
θ_2	-2.00	2064	-1.98	-1.99	0.41	0.96	2240	-1.99	-1.99	0.43	0.95	2249	-1.95	-1.98	0.47	0.96
	-1.50	2156	-1.48	-1.48	0.42	0.96	2245	-1.49	-1.50	0.44	0.96	2248	-1.45	-0.48	0.50	0.96
	-1.00	2199	-0.99	-0.99	0.43	0.95	2246	-1.00	-1.00	0.46	0.95	2245	-0.94	-0.96	0.53	0.95
	-0.50	2232	-0.46	-0.47	0.44	0.95	2249	-0.46	-0.49	0.47	0.95	2238	-0.43	-0.45	0.53	0.96
	0.00	2246	0.03	0.02	0.47	0.94	2250	0.04	0.02	0.49	0.94	2222	0.07	0.02	0.60	0.95
	0.50	2250	0.54	0.50	0.47	0.95	2250	0.56	0.54	0.51	0.95	2207	0.61	0.56	0.66	0.96
	1.00	2250	1.01	0.98	0.51	0.95	2248	1.05	1.01	0.55	0.95	2146	1.07	1.00	0.65	0.97
	1.50	2250	1.54	1.51	0.54	0.95	2247	1.57	1.51	0.58	0.95	2069	1.56	1.50	0.72	0.96
	2.00	2248	2.05	2.00	0.58	0.95	2239	2.08	2.02	0.62	0.96	1993	2.01	1.97	0.76	0.96

Note. Results are averaged over trait combinations with the same trait level. Number of converged replications refers to those of the 2250 response patterns that were non-homogeneous and yielded test information matrices of full rank.

ability and the effective speed of the test takers by maximum likelihood estimation. For the item parameters, we used the quasi-maximum likelihood estimates determined before. We first considered the estimates of effective ability as a proxy of the capability of the test takers. We then used the estimated effective ability and speed. We rotated both trait estimates and matched the first rotated trait with the capability values of the test takers. The rotated trait was then likewise used as a proxy. For both proxies, we determined the bias and the mean square error of estimation. Bias (dotted line) and mean squared error of estimation (straight line) is given for the true model (red line) and the 2-PL model (back line) in Figure S3.1 as a function of the true level of latent capability. Figure S3.2 visualizes the bias (dotted line) and mean square error (straight)

Table S3.2

True Value (TV), Number of Converged Replications (N), Average Estimate (M), Median Estimate (MD), Standard Error of Estimation (SE) and Coverage Frequency (CI) of Confidence Intervals with Confidence Level C=0.95 ($\alpha = 0.05$) of the Traits of the Linear Ballistic Accumulator Model A for Different Variants of the Model and a Test of 48 Items.

		Model A1					Model A2					Model A3				
TR	TV	N	M	MD	SE	CI	N	M	MD	SE	CI	N	M	MD	SE	CI
θ_1	-2.00	2209	-2.07	-2.01	0.58	0.95	2249	-2.31	-2.02	1.62	0.95	2250	-2.03	-1.97	0.75	0.95
	-1.50	2239	-1.57	-1.51	0.54	0.96	2250	-1.73	-1.49	1.47	0.95	2250	-1.53	-1.48	0.63	0.95
	-1.00	2247	-1.06	-1.01	0.49	0.95	2249	-1.14	-1.00	1.10	0.96	2250	-1.02	-0.99	0.59	0.95
	-0.50	2250	-0.53	-0.51	0.43	0.95	2250	-0.58	-0.52	0.76	0.95	2250	-0.51	-0.46	0.54	0.95
	0.00	2250	-0.03	-0.03	0.39	0.95	2250	-0.05	0.00	0.56	0.96	2248	-0.01	0.02	0.49	0.94
	0.50	2250	0.49	0.50	0.36	0.95	2250	0.45	0.49	0.54	0.95	2248	0.49	0.51	0.47	0.94
	1.00	2250	0.98	0.99	0.34	0.95	2250	0.99	0.99	0.39	0.95	2241	0.98	1.01	0.43	0.95
	1.50	2250	1.50	1.50	0.33	0.95	2250	1.49	1.50	0.36	0.95	2221	1.48	1.49	0.39	0.94
	2.00	2250	1.99	2.00	0.32	0.95	2250	1.99	2.00	0.34	0.95	2186	1.99	2.01	0.37	0.95
θ_2	-2.00	2214	-1.99	-1.98	0.30	0.95	2248	-2.00	-1.99	0.31	0.96	2250	-1.98	-1.98	0.32	0.96
	-1.50	2235	-1.50	-1.50	0.31	0.94	2250	-1.49	-1.50	0.30	0.96	2250	-1.46	-1.46	0.35	0.95
	-1.00	2247	-1.00	-1.01	0.31	0.94	2250	-0.98	-0.99	0.32	0.95	2250	-0.96	-0.98	0.37	0.95
	-0.50	2250	-0.50	-0.50	0.31	0.96	2250	-0.49	-0.51	0.33	0.95	2250	-0.46	-0.48	0.39	0.96
	-0.00	2250	0.00	0.00	0.32	0.95	2250	0.01	0.00	0.34	0.95	2250	0.06	0.03	0.45	0.95
	0.50	2249	0.51	0.50	0.33	0.95	2250	0.52	0.51	0.36	0.95	2247	0.56	0.52	0.46	0.96
	1.00	2250	1.02	1.01	0.34	0.96	2250	1.02	1.01	0.38	0.94	2232	1.05	1.03	0.50	0.95
	1.50	2250	1.53	1.53	0.38	0.95	2250	1.53	1.52	0.40	0.95	2220	1.55	1.51	0.55	0.95
	2.00	2250	2.04	2.02	0.40	0.95	2250	2.02	2.00	0.43	0.95	2195	2.06	2.00	0.59	0.95

Note. Results are averaged over trait combinations with the same trait level. Number of converged replications refers to those of the 2250 response patterns that were non-homogeneous and yielded test information matrices of full rank.

for the true model (red line) and the rotated trait estimate from the 2-PL model and the factor model (black line). The lines were determined by a kernel smoother.

The 2-PL model cannot recover the latent capability of the test takers. The estimates derived with the 2-PL model have a large bias. The mean square error is higher when the 2-PL model is used than when the data generating model is used for estimation. This suggests that the 2-PL model alone cannot replace the race model as a measurement model. The rotated trait estimates, on the other hand, are often at least as good as the estimates from the data generating model. Although the estimates tend to have a larger bias, they also have the smaller standard error of estimation. This results in a mean square error that is sometimes lower than that of the estimates from

Table S3.3

True Value (TV), Number of Converged Replications (N), Average Estimate (M), Median Estimate (MD), Standard Error of Estimation (SE) and Coverage Frequency (CI) of Confidence Intervals with Confidence Level C=0.95 ($\alpha = 0.05$) of the Traits of the Linear Ballistic Accumulator Model B for Different Variants of the Model and a Test of 24 Items.

		Model B1					Model B2					Model B3				
TR	TV	N	M	MD	SE	CI	N	M	MD	SE	CI	N	M	MD	SE	CI
θ_1	-2.00	3204	-1.94	-1.89	0.73	0.96	3688	-2.67	-2.12	2.14	0.96	3720	-2.33	-2.20	1.03	0.97
	-1.50	3512	-1.52	-1.48	0.70	0.97	3824	-2.12	-1.61	2.00	0.97	3851	-1.86	-1.72	1.03	0.97
	-1.00	3764	-1.05	-1.00	0.66	0.96	3916	-1.52	-1.11	1.86	0.97	3961	-1.39	-1.24	1.03	0.97
	-0.50	3905	-0.58	-0.53	0.65	0.97	3990	-0.92	-0.58	1.63	0.97	4001	-0.87	-0.72	1.00	0.97
	0.00	3968	-0.06	-0.01	0.61	0.96	4014	-0.30	-0.07	1.32	0.97	4021	-0.33	-0.17	0.94	0.97
	0.50	4002	0.43	0.48	0.56	0.96	4016	0.33	0.44	0.92	0.96	4018	0.18	0.33	0.91	0.97
	1.00	4004	0.95	0.97	0.52	0.96	3999	0.91	0.96	0.68	0.96	4000	0.70	0.85	0.85	0.97
	1.50	3992	1.47	1.50	0.49	0.95	3968	1.45	1.49	0.53	0.96	3978	1.22	1.35	0.83	0.97
	2.00	3950	1.98	1.99	0.46	0.96	3938	1.95	1.98	0.53	0.95	3932	1.74	1.86	0.79	0.97
θ_2	-2.00	3368	-1.98	-2.01	0.45	0.94	3923	-1.95	-2.00	0.46	0.94	3961	-1.98	-2.04	0.47	0.92
	-1.50	3650	-1.46	-1.53	0.55	0.93	3941	-1.45	-1.53	0.58	0.94	3963	-1.48	-1.56	0.58	0.91
	-1.00	3812	-0.94	-1.05	0.72	0.91	3932	-0.93	-1.07	0.75	0.92	3962	-1.00	-1.08	0.69	0.89
	-0.50	3886	-0.39	-0.56	0.92	0.90	3942	-0.43	-0.60	0.92	0.89	3974	-0.54	-0.63	0.83	0.87
	0.00	3931	0.10	-0.12	1.07	0.88	3967	0.03	-0.16	1.06	0.86	3965	-0.14	-0.23	0.95	0.84
	0.50	3961	0.58	0.33	1.27	0.84	3985	0.45	0.25	1.21	0.83	3975	0.18	0.10	1.06	0.81
	1.00	3973	0.89	0.65	1.38	0.81	3964	0.76	0.57	1.36	0.80	3965	0.43	0.38	1.16	0.77
	1.50	3956	1.18	0.92	1.50	0.78	3958	0.98	0.79	1.44	0.75	3968	0.56	0.53	1.23	0.71
	2.00	3764	1.46	1.31	1.53	0.76	3741	1.21	1.04	1.48	0.73	3749	0.80	0.75	1.25	0.71
θ_3	-2.00	3966	-2.90	-2.20	2.31	0.96	4032	-2.93	-2.25	2.32	0.96	4033	-2.32	-2.18	0.98	0.96
	-1.50	3964	-2.26	-1.67	2.15	0.96	4032	-2.32	-1.71	2.16	0.96	4037	-1.88	-1.66	0.93	0.97
	-1.00	3933	-1.69	-1.16	1.97	0.96	4032	-1.70	-1.19	1.97	0.97	4042	-1.33	-1.16	0.93	0.97
	-0.50	3929	-1.14	-0.66	1.87	0.97	4016	-1.12	-0.65	1.87	0.97	4043	-0.82	-0.67	0.89	0.97
	0.00	3885	-0.53	-0.16	1.67	0.97	3992	-0.53	-0.15	1.69	0.97	4028	-0.29	-0.15	0.83	0.97
	0.50	3818	0.10	0.37	1.43	0.96	3963	0.13	0.39	1.38	0.97	3997	0.24	0.36	0.79	0.97
	1.00	3762	0.66	0.88	1.32	0.96	3894	0.68	0.90	1.27	0.97	3920	0.78	0.89	0.73	0.97
	1.50	3624	1.23	1.39	1.18	0.97	3818	1.27	1.40	0.97	0.97	3791	1.27	1.36	0.70	0.96
	2.00	3420	1.79	1.90	0.97	0.97	3574	1.81	1.90	0.86	0.96	3591	1.79	1.87	0.65	0.96

Note. Results are averaged over trait combinations with the same trait level. Number of converged replications refers to those of the 4050 response patterns that were non-homogeneous and yielded test information matrices of full rank.

the data generating model. This suggests that an optimal combination of the responses and the response time is as good as a more sophisticated estimator of a test taker's capability. The knowledge how the responses and the response times should be combined, however, is knowledge that is not available in practice, unless the more

Table S3.4

True Value (TV), Number of Converged Replications (N), Average Estimate (M), Median Estimate (MD), Standard Error of Estimation (SE) and Coverage Frequency (CI) of Confidence Intervals with Confidence Level C=0.95 ($\alpha = 0.05$) of the Traits of the Linear Ballistic Accumulator Model B for Different Variants of the Model and a Test of 48 Items.

		Model B1					Model B2					Model B3				
TR	TV	N	M	MD	SE	CI	N	M	MD	SE	CI	N	M	MD	SE	CI
θ_1	-2.00	3710	-2.04	-2.00	0.60	0.96	3949	-2.28	-2.11	2.07	0.95	3976	-2.22	-2.13	0.79	0.96
	-1.50	3907	-1.55	-1.50	0.56	0.96	4010	-2.01	-1.56	1.80	0.95	4015	-1.72	-1.62	0.76	0.96
	-1.00	3989	-1.07	-1.03	0.52	0.96	4031	-1.36	-1.06	1.47	0.96	4029	-1.20	-1.12	0.72	0.96
	-0.50	4024	-0.56	-0.52	0.47	0.96	4036	-0.75	-0.55	1.18	0.96	4046	-0.67	-0.59	0.65	0.96
	0.00	4032	-0.04	-0.01	0.43	0.95	4038	-0.14	-0.03	0.90	0.96	4046	-0.18	-0.10	0.63	0.96
	0.50	4031	0.47	0.49	0.38	0.95	4034	0.44	0.48	0.53	0.95	4039	0.33	0.41	0.60	0.95
	1.00	4025	0.98	1.00	0.36	0.95	4028	0.95	0.97	0.44	0.95	4034	0.84	0.92	0.57	0.95
	1.50	4016	1.49	1.50	0.33	0.95	4009	1.47	1.49	0.35	0.95	4023	1.38	1.44	0.51	0.96
	2.00	3996	1.99	2.00	0.32	0.95	3993	1.98	1.98	0.34	0.95	4000	1.90	1.94	0.47	0.95
θ_2	-2.00	3773	-1.99	-2.01	0.27	0.94	4022	-1.98	-2.00	0.29	0.94	4034	-1.99	-2.02	0.34	0.92
	-1.50	3935	-1.49	-1.52	0.37	0.93	4019	-1.48	-1.52	0.40	0.93	4031	-1.48	-1.53	0.45	0.92
	-1.00	3977	-0.96	-1.03	0.55	0.93	4028	-0.97	-1.04	0.53	0.92	4034	-0.99	-1.05	0.55	0.91
	-0.50	4016	-0.43	-0.54	0.71	0.92	4028	-0.43	-0.55	0.73	0.91	4029	-0.49	-0.57	0.69	0.90
	-0.00	4033	0.13	-0.05	0.92	0.90	4021	0.09	-0.06	0.92	0.89	4034	-0.02	-0.13	0.83	0.88
	0.50	4028	0.60	0.39	1.07	0.88	4037	0.55	0.36	1.07	0.87	4029	0.37	0.27	0.97	0.84
	1.00	4032	1.02	0.81	1.23	0.84	4027	0.96	0.76	1.25	0.83	4034	0.68	0.62	1.08	0.81
	1.50	4021	1.33	1.13	1.35	0.80	4021	1.20	1.04	1.33	0.78	4022	0.90	0.84	1.19	0.76
	2.00	3915	1.59	1.41	1.44	0.77	3925	1.40	1.24	1.41	0.75	3961	1.04	0.97	1.24	0.72
θ_3	-2.00	4032	-2.63	-2.11	1.93	0.96	4042	-2.61	-2.09	1.97	0.95	4045	-2.19	-2.10	0.73	0.96
	-1.50	4018	-2.03	-1.61	1.72	0.96	4043	-2.00	-1.59	1.74	0.96	4045	-1.65	-1.57	0.65	0.96
	-1.00	4025	-1.44	-1.10	1.58	0.96	4042	-1.44	-1.11	1.57	0.95	4043	-1.16	-1.08	0.63	0.96
	-0.50	4003	-0.89	-0.59	1.45	0.96	4044	-0.85	-0.57	1.39	0.96	4043	-0.66	-0.58	0.59	0.95
	0.00	3998	-0.25	-0.08	1.13	0.96	4035	-0.25	-0.07	1.12	0.96	4047	-0.15	-0.08	0.57	0.96
	0.50	3975	0.31	0.45	0.97	0.96	4037	0.30	0.43	0.95	0.96	4041	0.37	0.43	0.52	0.96
	1.00	3939	0.87	0.94	0.70	0.96	4017	0.85	0.95	0.82	0.96	4031	0.92	0.95	0.43	0.95
	1.50	3916	1.40	1.45	0.58	0.96	3968	1.41	1.46	0.58	0.96	3986	1.43	1.46	0.41	0.96
	2.00	3824	1.93	1.95	0.48	0.96	3900	1.93	1.95	0.41	0.95	3927	1.93	1.95	0.35	0.96

Note. Results are averaged over trait combinations with the same trait level. Number of converged replications refers to those of the 4050 response patterns that were non-homogeneous and yielded test information matrices of full rank.

complex models have been fit. The combination of large bias and low standard error of estimation also has the effect that confidence intervals will not adhere to the intended coverage frequency.

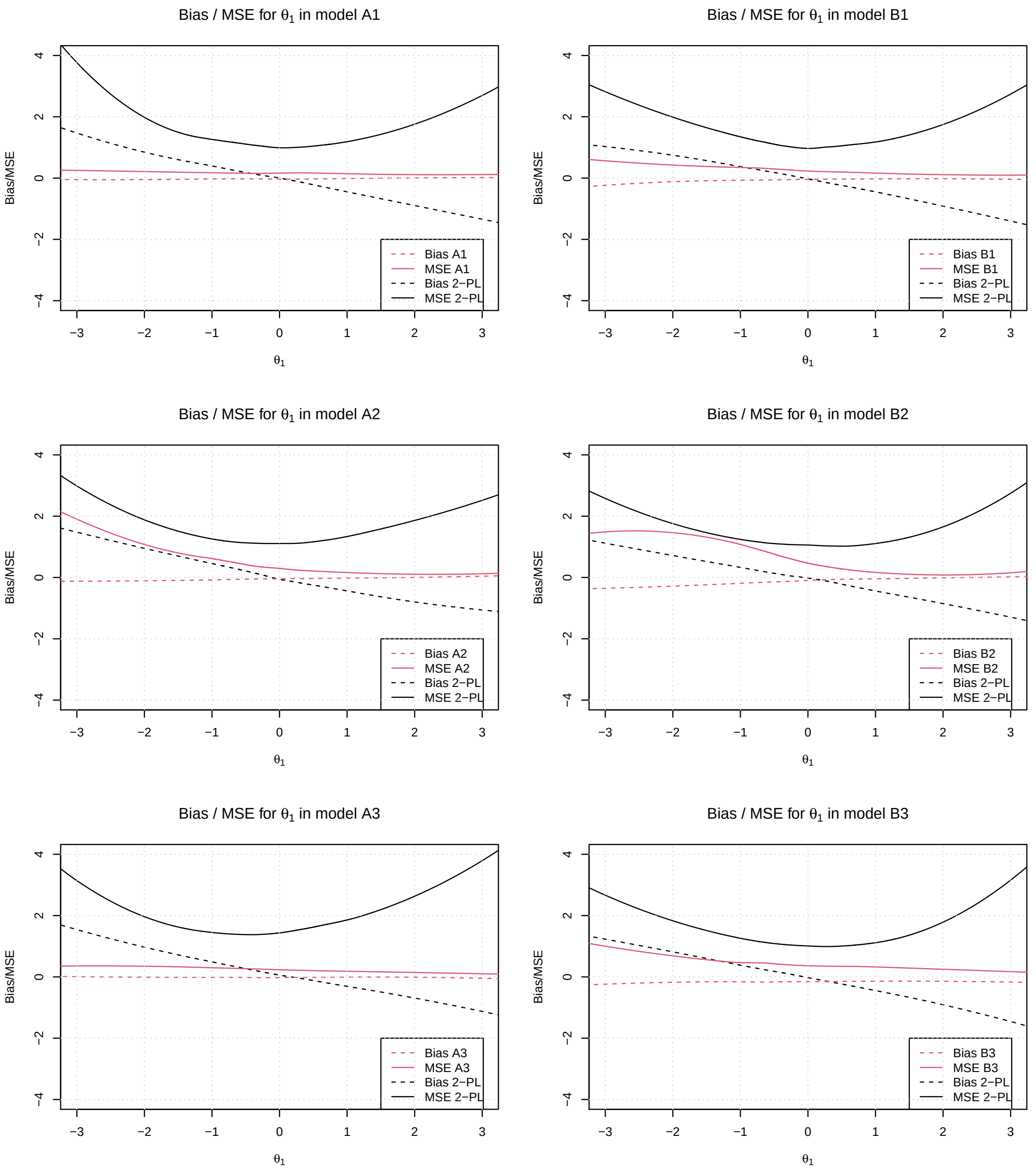

Figure S3.1. *Bias (dotted lines) and Mean Square Error (straight lines) for trait estimates of capability derived with the 2-PL model (black line) and the data generating model (red line) for the different models (Model A1–B3).*

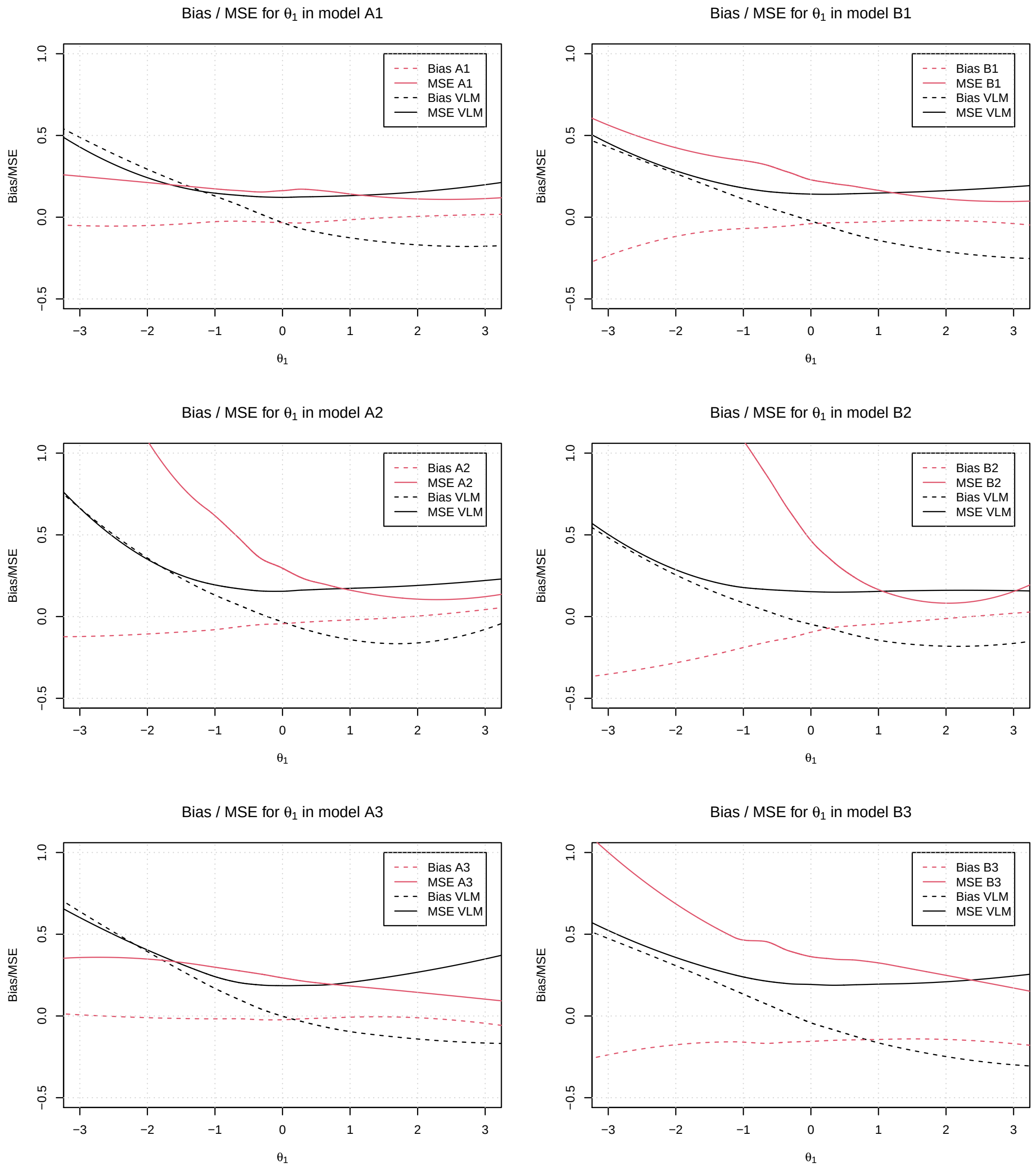

Figure S3.2. *Bias (dotted lines) and Mean Square Error (straight lines) for trait estimates of capability derived with a combination of the 2-PL model and the log-normal factor model (black line) and the data generating model (red line) for the different models (Model A1–B3).*

Supplement S4: Parameter Estimates and Results on Model Fit

The IMak data set was analyzed with Model B2M in the main document. The B2M model is a model with three accumulators representing progress, misinformation and disengagement and a normal mixture for the distribution of persistence. The marginal maximum likelihood estimates are given in Table S4.1 and Table S4.2. Table S4.1 contains estimates of the item parameters of the linear accumulators, namely the intercept, the discrimination coefficient and the standard deviation; see Equation 1, Equation 3 and Equation 6. We also report the standard error of estimation in brackets. Table S4.2 contains the estimates of the remaining parameters. These are the common guessing probability, the correlation coefficients of the latent traits and the parameters of the normal mixture distribution of persistence. The normal mixture has five parameters, two location parameters (μ_{c1},μ_{c2}), two scale parameters (σ_{c1},σ_{c2}) and a mixture proportion (π_c). Standard errors are reported in brackets.

We carefully assessed the fit of the B2M model to the IMak data. We compared the empirical quantiles to the ones implied by the model and the estimated item parameters, the recovery of the correlations between the responses and the response times, determined QQ-plots and the conditional accuracy functions. Here, we just report the QQ-plots and the conditional accuracy functions. Figure S4.1, visualizes the empirical quantiles (dotted line) and the theoretical quantiles (straight line) for different values as a function of α for correct (black dots) and incorrect responses (red dots). Note that empirical and theoretical quantiles are similar in most items. This suggests that Model B2M is capable to represent the conditional distribution of the response times. In some items like for example item 3, however, the fit is not optimal.

Figure S4.2 visualizes the conditional accuracy function. The conditional accuracy function maps the time spent on an item to the conditional solution probability. Empirical conditional accuracies (red dot) were determined as the average solution frequency of test takers with a response time within two response time quantiles. For the implied conditional accuracies (black dot), we used the solution probability implied by the model and the estimated item parameters. Conditional accuracies are based on

Table S4.1

Maximum likelihood estimates and standard error of estimation for the parameters of the linear accumulators in Model B2M and the IMak data

Item	α_0	α_1	σ_1	γ_0	γ_1	σ_3	β_0	β_1	σ_2
1	0.10	0.43	0.42	0.09	0.44	0.52	-2.00	0.46	1.09
	(0.03)	(0.04)	(0.02)	(0.08)	(0.07)	(0.05)	(4.25)	(1.68)	(1.50)
2	-0.33	0.47	0.55	-0.20	0.55	0.59	-1.03	0.51	0.73
	(0.05)	(0.06)	(0.04)	(0.11)	(0.08)	(0.05)	(0.55)	(0.29)	(0.20)
3	0.43	0.42	0.46	-0.75	0.83	0.53	-0.83	0.08	0.65
	(0.03)	(0.03)	(0.02)	(0.22)	(0.13)	(0.07)	(0.25)	(0.17)	(0.11)
4	0.75	0.35	0.35	-0.27	0.68	0.68	-0.66	0.13	0.83
	(0.02)	(0.03)	(0.02)	(0.23)	(0.13)	(0.11)	(0.52)	(0.16)	(0.28)
5	0.11	0.50	0.40	-0.75	0.83	0.54	-0.79	0.06	0.71
	(0.03)	(0.03)	(0.02)	(0.18)	(0.12)	(0.07)	(0.21)	(0.11)	(0.11)
6	-0.49	0.49	0.47	-0.21	0.69	0.44	-3.22	1.26	1.36
	(0.04)	(0.05)	(0.03)	(0.04)	(0.04)	(0.02)	(2.23)	(0.87)	(0.82)
7	0.18	0.46	0.38	-0.52	0.82	0.32	-1.24	0.52	1.13
	(0.03)	(0.03)	(0.02)	(0.09)	(0.07)	(0.04)	(0.45)	(0.16)	(0.33)
8	-0.50	0.48	0.43	-0.33	0.67	0.44	-2.71	1.59	1.24
	(0.04)	(0.04)	(0.03)	(0.05)	(0.04)	(0.02)	(1.17)	(0.70)	(0.39)
9	-0.07	0.44	0.42	-0.20	0.68	0.49	-4.67	2.81	1.29
	(0.03)	(0.04)	(0.02)	(0.05)	(0.05)	(0.03)	(3.05)	(1.59)	(0.54)
10	-0.83	0.55	0.46	-0.33	0.72	0.39	-2.61	1.87	1.22
	(0.04)	(0.06)	(0.03)	(0.04)	(0.04)	(0.02)	(0.81)	(0.53)	(0.23)
11	-0.75	0.51	0.47	-0.56	0.75	0.44	-1.36	1.63	0.90
	(0.04)	(0.05)	(0.03)	(0.06)	(0.06)	(0.03)	(0.33)	(0.29)	(0.09)
12	-1.20	0.54	0.51	-0.46	0.67	0.47	-1.64	1.90	0.89
	(0.06)	(0.07)	(0.04)	(0.05)	(0.05)	(0.03)	(0.42)	(0.32)	(0.11)
13	-0.19	0.42	0.33	-0.40	0.51	0.50	-1.20	1.83	0.76
	(0.03)	(0.03)	(0.02)	(0.07)	(0.07)	(0.05)	(0.35)	(0.31)	(0.06)
14	-0.61	0.41	0.45	-0.37	0.62	0.44	-1.45	2.00	0.71
	(0.04)	(0.05)	(0.03)	(0.05)	(0.05)	(0.03)	(0.34)	(0.28)	(0.07)
15	-0.86	0.50	0.49	-0.81	0.77	0.52	-0.83	1.74	0.56
	(0.04)	(0.06)	(0.03)	(0.09)	(0.11)	(0.05)	(0.20)	(0.18)	(0.06)
16	-0.74	0.60	0.43	-0.76	0.77	0.58	-0.48	1.63	0.61
	(0.05)	(0.05)	(0.03)	(0.13)	(0.12)	(0.07)	(0.19)	(0.17)	(0.05)
17	-0.60	0.46	0.44	-0.79	0.86	0.58	-0.49	1.63	0.70
	(0.04)	(0.05)	(0.03)	(0.13)	(0.14)	(0.10)	(0.20)	(0.19)	(0.06)

quantiles from $q_{0.10}$, $q_{0.20}$ to $q_{1.00}$. Note that the conditional accuracies are an increasing function of response time in most items. This suggests that spending more time on the task increases the probability to give a correct response. There, however, seems to be an upper limit which bounds the effect of time. Empirical and implied conditional accuracy functions are generally in good agreement.

Table S4.2

Maximum likelihood estimates and standard error of estimation for the parameters of Model B2M that are not related to the accumulators

π_g	ρ_{12}	ρ_{13}	ρ_{23}	μ_{c1}	σ_{c1}	μ_{c2}	σ_{c2}	π_c
0.19	0.03	0.49	0.76	-1.64	0.60	0.14	0.90	0.07
(0.02)	(0.08)	(0.05)	(0.06)	(0.18)	(0.18)	(0.04)	(0.04)	(0.02)

Note. π_g: Guessing Probability; ρ_{12}–ρ_{23}: Correlation of Traits;
μ_{c1}–π_c: Parameters of Normal Mixture Distribution

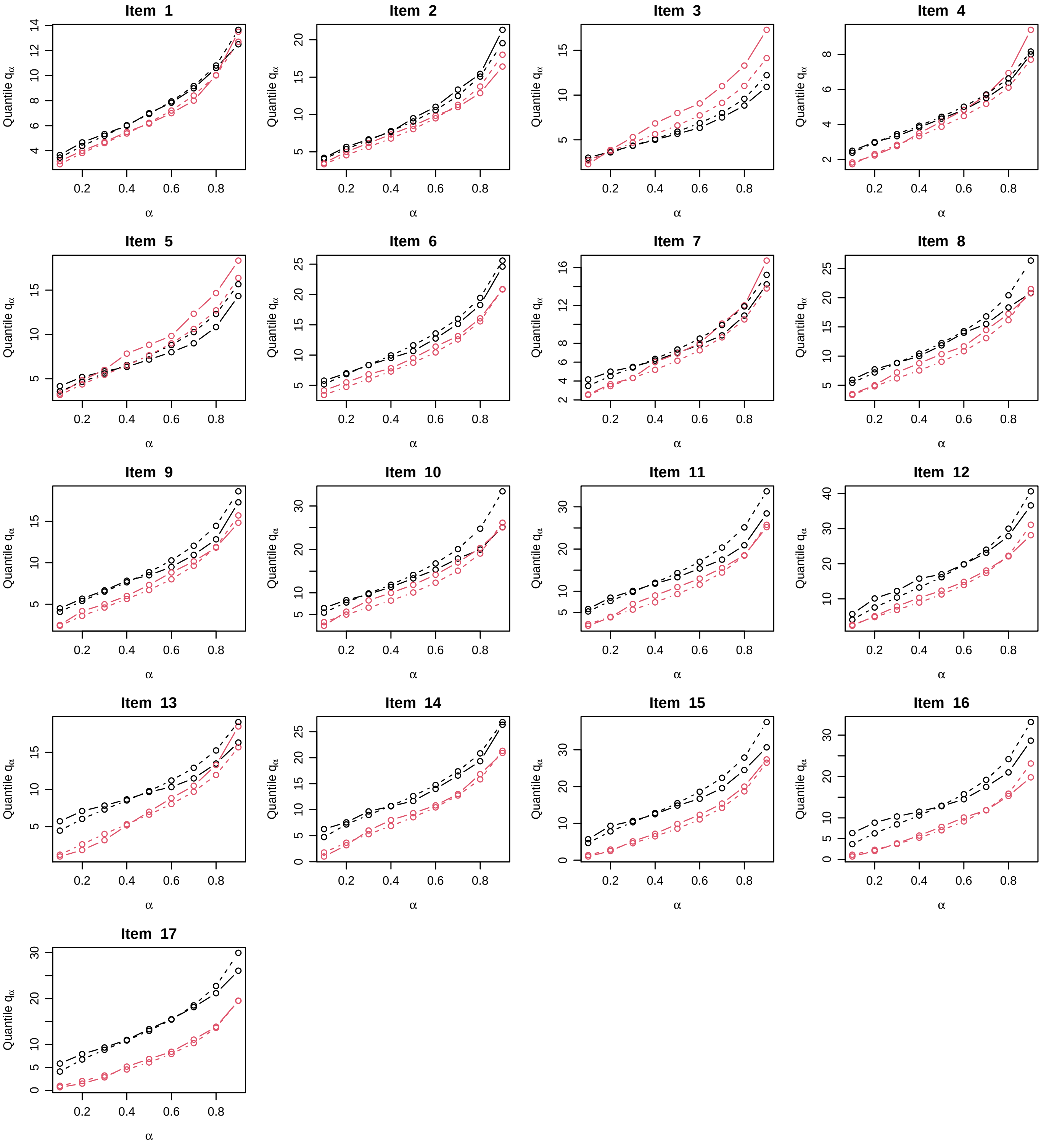

Figure S4.1. *Empirical Quantiles (Straight Line) as well As Implied Quantiles (Dotted Line) for Response Times in Correct (Black) and Incorrect (Red) Responses for the Items of the IMak Data and Model B2M.*

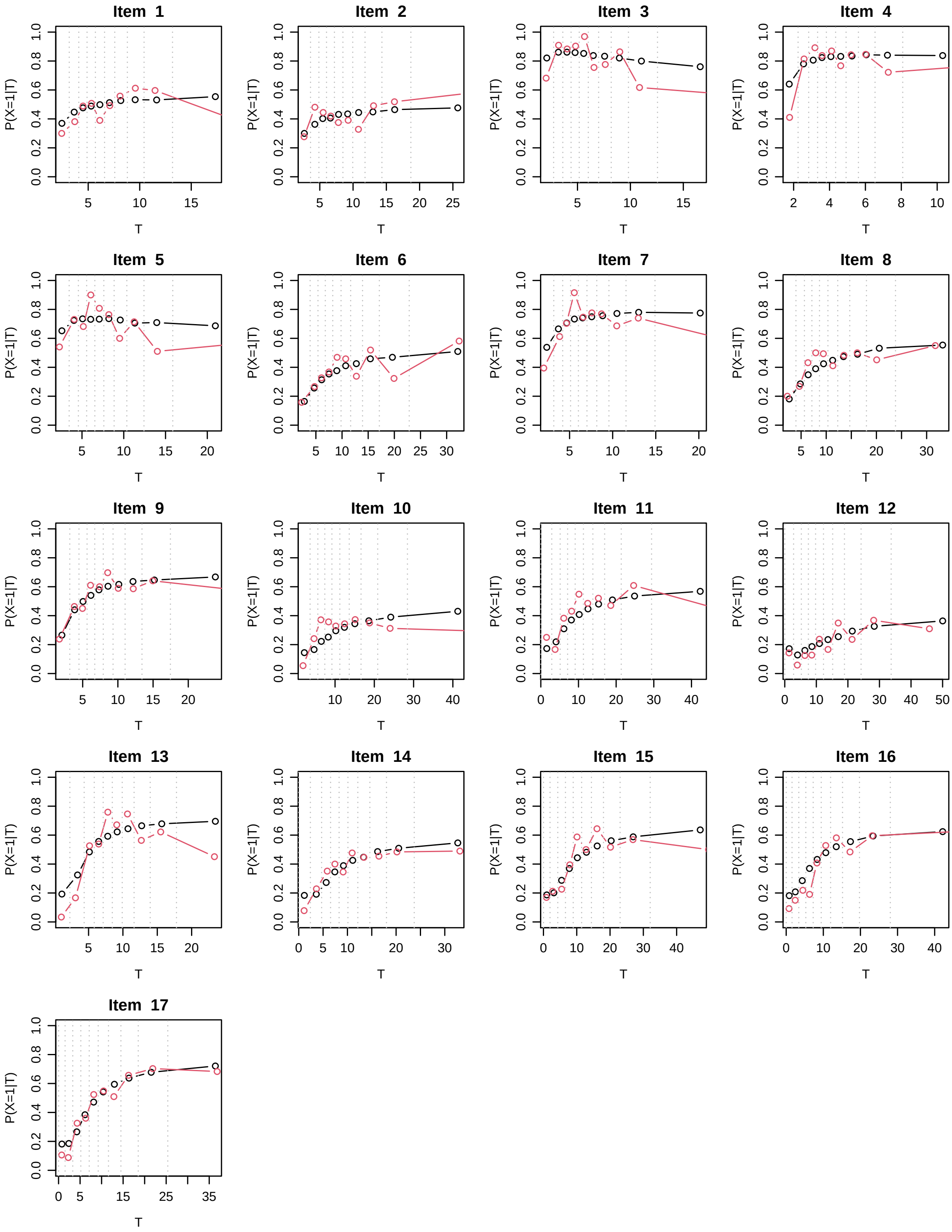

Figure S4.2. *Empirical Conditional Accuracy Function (Red) and Conditional Accuracy Function Implied by Model B2M and the Parameter Estimates (Black) for the Items of the IMak Data.*
